# Effects of Ultrasound-Assisted Extraction of Biofractions from Yellow Mealworm Larvae Fed a Probiotic-Enriched Diet

**DOI:** 10.3390/insects17090919

**Published:** 2026-09-02

**Authors:** Sabina Dahal, Aberham Hailu Feyissa, Lucas Sales Queiroz, Antoine Lecocq, Heidi Olander Petersen, Charlotte Jacobsen, Uri Lesmes, Federico Casanova

**Affiliations:** 1 Food Production Engineering Research Group (FPE), National Food Institute, Technical University of Denmark (DTU), 2800 Kongens Lyngby, Denmark; sabda@food.dtu.dk (S.D.); abhfe@food.dtu.dk (A.H.F.); lusaqu@food.dtu.dk (L.S.Q.); hope@food.dtu.dk (H.O.P.); 2 Section of Organismal Biology, Department of Plant and Environmental Sciences (PLEN), University of Copenhagen, 1172 Frederiksberg, Denmark; antoine@plen.ku.dk; 3 Research Group for Bioactives–Analysis and Application, National Food Institute, Technical University of Denmark(DTU), 2800 Kongens Lyngby, Denmark; chja@food.dtu.dk; 4 Lab of Food Chemistry, Bioactives and Digestion, Faculty of Biotechnology and Food Engineering, Technion Faculty of Biotechnology and Food Engineering, Israel Institute of Technology, Haifa 3200003, Israel; lesmesu@bfe.technion.ac.il

**Keywords:** yellow mealworm, probiotics, ultrasound-assisted extraction, *Tenebrio molitor* larvae protein, functional properties

## Abstract

The increasing demand for sustainable protein sources has stimulated interest in edible insects such as *Tenebrio molitor* (yellow mealworm) larvae. While probiotics supplementation has been explored to improve insect growth performance, its impact on protein quality and downstream processing remains poorly understood. This study investigated the combined effects of probiotic-enriched diet supplementation during larval rearing and post-harvest ultrasound-assisted extraction (UST) on the protein and lipid fractions of yellow mealworm larvae. Three probiotic strains, namely, *Pediococcus pentosaceus* (PP), *Bacillus velezensis* (BV), and *Bacillus coagulans* (BC), were incorporated into larval diets and compared with a standard diet (without probiotics). Protein extraction was performed by both alkaline solubilization–isoelectric precipitation, which served as a control, and ultrasound application for 5, 15, and 25 min. The results from the trial found effects of both probiotics and UST, as well as their interaction, on the protein yield, although an improvement in yield was not obtained with increased sonication, and the BV group produced the highest yield among the probiotic treatment groups. In addition to this, the study did not find any effects associated with ultrasound pretreatment or probiotics on proximate composition and color, except fat % and increased lipid co-extraction, especially at longer durations. Probiotics, on the other hand, showed an influence on the fatty and amino acid profile, while they interacted with UST to affect the extracts’ content. This study did not find any effects on particle size, but the zeta potential was affected by probiotic diet enrichment. Similarly, the free SH content was influenced by sonication, while both probiotics and UST played a role in hydrophobicity. Regarding the solubility, it was found to be pH-dependent and ranged from 0 to ≈90%. Importantly, this study found that probiotics did not have any effect on protein solubility; however, UST had an effect at pH 2 and 7 but not at pH 11. In terms of hydrophobicity, both factors and their interactions were found to have an effect. Moreover, this study could not determine any effects on the inherent fluorescence absorbance of protein molecules. Apart from this, FTIR analysis revealed β-sheet-dominated secondary structures (≈41–47%), with treatment-dependent shifts in α-helix and β-turn contributions. The heat enthalpies associated with denaturation and melting temperature were influenced by sonication, where probiotics could not confer an effect. Overall, this study might demonstrate the strain-specific improvement in protein yield and modulation of the physicochemical functionality, as well as the nutritive value of the protein. Both biological factors and ultrasound-assisted extraction processes can act together to tailor the physicochemical and functional properties of biofractions, but more studies are needed for process optimization.

## Simple Summary

Insects are widely studied as a prominent source of high-quality nutritional food. Studies have been done extracting protein from yellow mealworm, as this protein is rich in essential amino acids required for balanced nutrition. Likewise, exploring the effects of a probiotic-enriched diet in insect farming is gaining interest among researchers seeking to understand its applications and benefits in mass production. There have been a limited number of studies done on understanding the role of feed and processing techniques in protein extraction, in addition to follow-up studies. This study tried to investigate the influence of probiotic feeding that was carried out during larval rearing and ultrasound (UST) pretreatment for biofraction extraction; this was followed by analysis of the protein’s physicochemical and functional traits. Notably, the study could not find any benefits of sonication in the protein yield when raw insects were used for processing without defatting. However, differences were observed in the yield according to probiotic group. Moreover, changes were observed in amino acid and fatty acid profiles, followed by changes in physicochemical and techno-functional properties. However, longer sonication did not show any beneficial effects on protein functionalities and use in food applications. Thus, this study demonstrated a strain-specific improvement in yield, and these biological factors, in combination with ultrasound-assisted extraction, modulate biofractions’ extraction and their physicochemical and functional properties.

## 1. Introduction

The global demand for high-quality protein continues to increase amid population growth and the associated pressure on finite natural resources [1,2]. Conventional livestock-based food systems are associated with substantial environmental impacts, including intensive land and water use and greenhouse gas emissions, which has intensified interest in alternative, and more sustainable, protein sources [3]. In this context, edible insects have emerged as a potential protein source due to their favorable nutritional profile and comparatively small environmental footprint [4].

Among edible insects, *Tenebrio molitor* (yellow mealworm) larvae are considered particularly suitable for large-scale production because of their short life cycle, ease of rearing, and high nutritional value, particularly as sources of protein and lipids [5]. Mealworm larvae typically contain 40–60% protein on a dry matter basis and exhibit a balanced essential amino acid profile comparable to those of conventional animal proteins [6,7]. In addition to protein, this insect species is a rich source of healthy fats, minerals, and other bioactive compounds, supporting its use as a raw material for food and feed ingredients. Despite these nutritional advantages, consumer acceptance of insects in Western diets remains limited. Processing insects into biofractions, such as protein isolates, lipid fractions, or powders, has been identified as an effective strategy for reducing sensory and visual barriers while facilitating the incorporation of the insects into familiar food matrices [8]. Moreover, compared with conventional protein sources, protein fractions extracted from insects often exhibit improved digestibility and techno-functional properties, including gelation, emulsification, and foaming, which are critical for food applications [9].

In parallel, probiotic supplementation has gained attention as a strategy for improving insect production, drawing from established practices in livestock and aquaculture [10]. Recent studies have evaluated the use of lactic acid bacteria and spore-forming *Bacillus* species as dietary supplements for insects and have reported beneficial effects, following successful gut establishment, on growth performance, survival, and biomass yield [10,11,12,13]. However, only a limited number of studies have reported changes in the biochemical composition of insects following the provision of probiotic-enriched diets [13,14,15]. Importantly, the consequences of probiotic feeding for downstream processing, particularly protein and lipid extraction efficiency and the techno-functional properties of the extracted proteins, remain poorly understood. Elucidating whether probiotic-induced physiological changes in larvae influence protein extractability and functionality is therefore relevant to ingredient development.

From a processing perspective, protein extraction efficiency and functional performance are strongly influenced by pretreatment and extraction conditions. In insects, proteins are embedded in complex matrices containing lipids, chitin, and structural components, which can limit extraction efficiency when conventional methods are applied [16]. Alkaline (pH-shift) extraction is widely used for insect protein recovery but is often associated with protein denaturation, undesirable color changes, high chemical consumption, and substantial wastewater generation [17]. These limitations have stimulated interest in alternative and more sustainable extraction approaches. Among the emerging technologies, ultrasound-assisted extraction (UST) has been identified as a promising method for insect protein recovery [18]. Ultrasound induces acoustic cavitation, resulting in microbubble formation and collapse, which disrupts cellular structures and enhances mass transfer, thereby improving extraction efficiency [19]. Several studies have shown that ultrasound-assisted alkaline extraction increases insect protein yield and improves techno-functional properties compared with conventional alkaline extraction [20,21,22,23,24]. In addition, ultrasound-assisted extraction has been reported to preserve or enhance the bioactivity of insect-derived compounds while reducing processing time and solvent consumption [21,22,24,25].

The objective of this study was to evaluate the combined effects of probiotic supplementation and the ultrasound-assisted extraction of protein and lipids from raw and frozen *T. molitor* larvae. Three probiotic strains, namely, *Pediococcus pentosaceus* (PP), *Bacillus velezensis* (BV), and *Bacillus coagulans* (BC), and three ultrasound treatment durations (5, 15, and 25 min) were investigated and compared with a control (Std.) diet and alkaline solubilization–isoelectric precipitation without ultrasound (0 min), which served as the control extraction method. The resulting protein powder (TMLP) and lipids extracted from the raw and frozen (minimally processed) larvae were characterized in terms of yield, biochemical and physicochemical composition, and the physicochemical and functional properties of the proteins.

## 2. Materials and Methods

### 2.1. Experimental Design

Three probiotic strains, namely, *P. pentosaceus* (PP), *B. velezensis* (BV), and *B. coagulans* (BC), were selected for the feeding of yellow mealworm larvae (TML) during rearing. Plain wheat bran served as the control (Std.) diet. Both the control and probiotic-enriched diets were designated as feed groups. Brewer’s spent grain (BSG) was provided as wet feed regardless of the treatment. The entirety of the larval rearing, feeding, and harvesting procedures were described by Dahal et al. [26]. Ultrasound pretreatment (UST) was applied for 5, 15, and 25 min, while isoelectric precipitation without ultrasound (0 min, iso) served as the control extraction. The experimental design combined a categorical factor (probiotics) and a quantitative factor (UST), each at four levels, with three replicates per treatment combination (2^4^).

### 2.2. Chemicals

Sodium hydroxide (NaOH, pellets), Sodium Bisulphite, and citric acid (crystals) were purchased from Sigma-Aldrich (Taufkirchen, Germany). Sodium dihydrogen phosphate dihydrate and disodium hydrogen phosphate dihydrate were obtained from Merck KGaA (Darmstadt, Germany). Reagents for protein quantification by the Dumas method (L-aspartic acid and starch flour) were sourced from Sigma-Aldrich (Tokyo, Japan) and Elementar Analysensysteme GmbH (Langenselbold, Germany). Protein electrophoresis reagents, including Precision Plus Protein™, All Blue pre-stained standards, 12% TGX Stain-Free polyacrylamide gels, 2× Laemmli sample buffer, and Coomassie Brilliant Blue R-250, were purchased from Bio-Rad (Mississauga, ON, Canada). Reagents for amino acid and fatty acid analysis and 8-anilino-1-naphthalenesulfonic acid (ANS) were obtained from Thermo Fisher Scientific and Sigma-Aldrich (Taufkirchen, Germany). Chemicals used for Ellman’s reagent preparation (glycine, Tris, and EDTA) were purchased from Sigma-Aldrich (Taufkirchen, Germany).

### 2.3. Protein and Fat Extraction

Frozen yellow mealworm (*T. molitor*) larvae were crushed into a paste using a kitchen grinder (Bodum^®^, Skandinavien A/S, Humlebæk, Denmark) and dispersed in distilled water at a mass-to-volume ratio of 1:3 (*w*/*v*), as previously described by Zhang et al. [25]. Since the larvae were not dried or defatted prior to protein extraction and were used in the raw and frozen state, they were designated as minimally processed larvae. Briefly, 33 g of larvae were weighed, and the volume was adjusted to 100 mL. Sodium bisulfite (1.0 g kg^−1^, based on insect weight) was added, and the dispersion was mixed at 500 rpm for 2 min using a mechanical stirrer (RCT digital, IKA, Staufen, Germany) at room temperature (25 °C).

For the control extraction, the suspension pH was adjusted to 10.0 ± 0.1 using 1.0 M NaOH and stirred at 500 rpm for 1 h. The mixture was centrifuged using a refrigerated centrifuge (Sigma 4-16KS, Sigma Laborzentrifugen GmbH, Osterode am Harz, Germany) at 5000× *g* for 20 min at 4 °C, and the supernatant was collected. Protein precipitation was induced by adjusting the pH to 4.3–4.5 with 1.0 M citric acid, followed by sedimentation for 1 h at 25 °C and centrifugation under the same conditions. The precipitate was washed three times and resuspended in distilled water, and the pH was adjusted to 7.0 with 1.0 M NaOH; this was followed by freeze-drying.

The freeze-dried protein samples were defatted using 96% ethanol at a 1:20 (*w*/*v*) ratio, followed by stirring at 500 rpm for 1 h at room temperature. The suspension was vacuum filtered (Whatman^®^, Cytiva, Dassel, Germany). The filtrate was then subjected to fractional distillation using a rotary evaporator (Laborota 4000-efficient, Heidolph Instruments GmbH & Co. KG, Schwabach, Germany) to separate the oil and recover ethanol for reuse.

Protein extraction with ultrasound pretreatment followed the same procedure as the control extraction, except that ultrasound was applied prior to pH adjustment to 10.0. The suspension was sonicated using a probe-type ultrasonic processor (Branson Sonifier, Emerson, St. Louis, MO, USA) operating at 20 kHz and 500 W, with 5 s on/off pulses, for 5, 15, or 25 min. All details regarding changes in temperature, acoustic power output, and energy intensity are provided in File S1. UST treatment was performed in a temperature-controlled cold-water bath, with the probe positioned at the center and approximately midway along the total sample height to ensure proper acoustic distribution throughout the sample volume. Protein extraction yield for both the control and ultrasound-assisted extractions was calculated according to Equation (1) [24].

(1) $$Protein\;yield(\%)=\frac{W_{f}\times C_{f}}{W_{i}\times C_{i}}\times 100$$

where *W_f_* and *C_f_* represent the dry weight (g) and protein concentration (%) of the final extract mass fraction, respectively, and *W_i_* and *C_i_* correspond to the dry weight (g) and protein concentration (%) of the whole raw larvae meal. Extraction yield and mass balance were determined for each treatment time and diet combination. Each experiment was carried out in three batches.

### 2.4. Biochemical and Physicochemical Properties of Extracted Proteins

#### 2.4.1. Proximate Composition

Proximate composition was determined for the protein extracts before and after defatting according to AOAC methods [26,27]. The protein content of the larvae samples was determined using the Dumas method (Elementar Analysensysteme GmbH, Langenselbold, Germany). Nitrogen-to-protein conversion factors of 4.76 and 5.6 were applied to the freeze-dried protein extracts before defatting and the defatted protein concentrates, respectively, to avoid overestimation of protein content [28]. Total fat content was measured using an ORACLE™ universal fat analyzer (CEM Corporation, Matthews, NC, USA).

#### 2.4.2. Amino Acid Composition

The total amino acid profile of the insect samples was determined by LC–MS, as described by Queiroz et al. [29]. Briefly, samples were subjected to acid hydrolysis using 6 M HCl (10 mg sample per mL HCl) at 110 °C for 18 h. After cooling to room temperature (25 °C), the hydrolysates were analyzed, both undiluted and after a threefold (1 + 2) dilution with 6 M HCl, to enable the quantification of both high- and low-abundance amino acids. Cysteine and tryptophan were not quantified because they were degraded during acid hydrolysis. The digested filtrate was filtered through a 0.2 μm PTFE syringe filter (Q-Max, Frisenette ApS, Knebel, Denmark).

A 100 μL aliquot of the filtered hydrolysate was pipetted into a 4 mL vial, and the pH was adjusted by adding 1.5 mL of 0.2 M KOH. An additional 1.6 mL of buffer (100 mM ammonium formate, pH 3.1) was added to the hydrolysate. Finally, 1 mL of the final hydrolysate was transferred to LC–MS vials and analyzed by LC–(APCI)–MS (Agilent Technologies, Santa Clara, CA, USA). The amino acids were identified using the LC–MS method, and quantification was performed using external standard calibration curves. The calibration curve included a standard mixture at 500 μmol L^−1^ (amino acid standard: Supelco 79248, 2.5 mM, 6.13). Details of the calibration curves for each amino acid are provided in File S2.

#### 2.4.3. SDS-PAGE

Protein profiles of TMLP were analyzed by SDS-PAGE according to Perez et al. [28], with minor modifications. Briefly, 50 mg of *T. molitor* larval protein was solubilized in 2 mL of 1% SDS solubilization buffer (pH 8.3) by shaking for 1 h, followed by brief Polytron homogenization. Samples were heated at 100 °C for 2 min, cooled, reheated, and centrifuged at 20,000× *g* for 15 min. Following TCA precipitation, the supernatant was dissolved in 2× Laemmli sample buffer and distilled water according to the protein concentration; this was followed by centrifugation at 30,000× *g* for 5 min. Protein extracts (10 µL) were separated on 15% TGX Stain-Free polyacrylamide gels using 1× Tris–glycine–SDS running buffer at 150 V and 90 mA for 1 h. Precision Plus Protein™ All Blue molecular weight markers (Bio-Rad Laboratories, Mississauga, ON, Canada) were included. Gels were stained with colloidal Coomassie Brilliant Blue and imaged for band analysis.

#### 2.4.4. Determination of Free Sulfhydryl (SH) Groups

The free sulfhydryl (SH) content of TMLP was measured according to the procedure described by Yousefi et al. [30], with slight modifications. To obtain a final protein concentration of 5 mg mL^−1^, 25 mg of insect protein powder was added to 5 mL of reagent buffer (0.09 M glycine, 0.086 M Tris, and 4 mM EDTA at pH 8.0). After adding 50 μL of Ellman’s reagent, the resulting suspensions were incubated for 1 h at room temperature (25 °C) with occasional agitation and then centrifuged for 15 min at 10,000× *g*. The absorbance of the supernatant was measured at 412 nm using a Hitachi U-1500 spectrophotometer (San Jose, CA, USA), with the reagent buffer used as the blank. The concentration of free SH groups was calculated in mmol SH g^−1^ using Equation (2).

(2) $$SH\left(µ\text{mol}\right)=\frac{\left(73.53\times A_{412}\times D\right)}{C}$$

where *A*_412_ expresses the absorbance at 412 nm and *D* the dilution factor; the factor 73.53 was derived from 10^4^/(1.36 × 10^2^), and C is the concentration of the dispersion sample (mg mL^−1^ mL^−1^). The molar absorptivity constant was 1.36 × 10^2^ m^−1^ cm^−1^ [30].

#### 2.4.5. Color Measurement

The color parameters (L*, a*, and b*) of TMLP were measured using a CR-400 chromameter (Konica Minolta, Tokyo, Japan) with illuminant D65 and an 8 mm aperture. The Lab* (CIELAB) system was used to characterize color, where L* refers to lightness (0–100), a* refers to the red/green axis (+a = red, −a = green), and b* refers to the yellow/blue axis (+b = yellow, −b = blue) [31]. Samples were evenly distributed on a Petri dish, and measurements were taken at three different surface positions. The instrument was calibrated using a white reference tile (L* = 97.58, a* = 5.60, b* = +7.57). Color values were reported in the CIELAB color space.

#### 2.4.6. Hydrophobicity

Surface hydrophobicity of TMLP was determined according to Pinel et al. [1], with minor modifications. Protein dispersions (5 mg mL^−1^) were prepared in 2 mM phosphate buffer (pH 7.0), stirred for 30 min at 500 rpm, and centrifuged at 4000× *g* for 10 min. The supernatants were diluted to protein concentrations ranging from 0.01 to 0.05% (*w*/*v*) to obtain a final volume of 2 mL. Subsequently, 5 mL of 8-anilino-1-naphthalenesulfonate (ANS) solution (8 mM), freshly prepared in 2 mM phosphate buffer under dark conditions, was prepared. In sum, 50 μL of the 8 mM ANS solution was added to each diluted sample. The samples were vortexed for 10 s and incubated in the dark for approximately 15 min before measurement. From each sample, 200 μL was transferred to a 96-well plate. Fluorescence was measured using a multimode microplate reader (BioTek Synergy H1, Agilent Technologies, USA) at excitation and emission wavelengths of 380 and 460 nm, respectively. Extrinsic fluorescence was obtained after correcting for intrinsic protein and buffer fluorescence. Surface hydrophobicity (H0) was calculated as the slope of fluorescence intensity versus protein concentration [1]. All experiments were performed in triplicate.

#### 2.4.7. Intrinsic Fluorescence

Intrinsic fluorescence of TMLP dispersions (1.0 mg mL^−1^) was measured using a spectrophotometer (F-4500, Hitachi, Tokyo, Japan) according to the methods described by Zhang et al. [31] and Boukil et al. [32], with slight modifications. Samples were excited at 295 nm, and emission spectra were recorded from 300 to 400 nm with excitation and emission slit widths of 10 nm and a scan rate of 240 nm min^−1^. The emission results were expressed as relative fluorescence intensity (RFI, a.u.).

#### 2.4.8. Protein Solubility

Protein solubility was determined using the Dumas method according to the process described by Gravel et al. [33]. Protein dispersions (5 mg mL^−1^) were prepared in 2 mM phosphate buffer, and the pH was adjusted to 2, 5, 7, and 11 using 1 M NaOH or citric acid (C_6_H_8_O_7_·H_2_O). After pH adjustment, the samples were stirred at 500 rpm for 1 h and centrifuged at 8000× *g* for 20 min at 20 °C. The supernatants were collected, and soluble protein content was determined by Dumas analysis, using a nitrogen-to-protein conversion factor of 6.25 [1]. Protein solubility was expressed as the percentage of soluble protein relative to the total protein content.

#### 2.4.9. Particle Size and ζ-Potential Measurement

Protein dispersions were prepared at a concentration of 1 mg mL^−1^ in 2 mM phosphate buffer (pH 7.5) and stirred at 500 rpm for 30 min. The suspensions were centrifuged at 15,000× *g* for 15 min at 20 °C (Oledich^®^ JE Centrifuge, Beckman Coulter, Palo Alto, CA, USA), and the supernatants were collected for analysis. Both particle size distribution and zeta potential were determined using a Zetasizer Nano (Malvern Panalytical, Malvern, UK). The refractive indices for the samples were set to 1.45 for the protein and 1.33 for the dispersant. Other test parameters were fixed as follows: scattering angle, 90°; equilibration time, 60 s; and test temperature, 5 °C. Particle size was expressed as D(3,2) and D(4,3), where D(3,2) represents the volume–surface mean diameter and D(4,3) represents the volume–mean diameter. All measurements were performed in triplicate.

#### 2.4.10. Fourier Transform Infrared Spectroscopy (FTIR)

The secondary structures of the TMLP samples were analyzed according to the method described by Queiroz et al. [29], with minor modifications. A PerkinElmer Spectrum 100 FT-IR spectrometer (Waltham, MA, USA), equipped with a Universal Attenuated Total Reflectance (UATR-FTIR) sensor, was used to record the FTIR spectra in transmission mode over a range of 4000–500 cm^−1^. Spectra were plotted as percentage transmittance (%T) as a function of wavenumber (cm^−1^). Signals were recorded using four scans. OriginPro 2023 software (OriginLab Corporation, Northampton, MA, USA) was used to calculate the second derivative of the signal in the Amide I region (1600–1700 cm^−1^) for secondary structure analysis [28]. All analyses were performed in triplicate.

#### 2.4.11. Differential Scanning Calorimetry (DSC)

Thermal stability was analyzed by differential scanning calorimetry (DSC) according to the process described in Pinel et al. [1], using a DSC 250 instrument (TA Instruments, New Castle, DE, USA) equipped with a refrigerated cooling system. Calibration was performed using distilled water (melting point 0 °C, ΔH = 334 J g^−1^) and indium (melting point 156.5 °C, ΔH = 28.5 J g^−1^). Samples (5–10 mg) were sealed in Tzero™ hermetic aluminum pans (20 µL) (TA instruments, Hüllhorst, Germany), with nitrogen used as the purge gas at 50 mL min^−1^. Samples were firstly cooled to 0 °C and subsequently heated from 20 to 150 °C at a rate of 1 °C min^−1^. Measurements were performed in duplicate. Thermograms (heat flow versus temperature) were analyzed using TRIOS^®^ software version 4.0 (TA Instruments, New Castle, DE, USA) to determine glass transition temperature (T_g_), denaturation temperature (T_d_), melting temperature (T_m_), and associated enthalpy change (ΔH).

### 2.5. Fatty Acid Profile

The fatty acid composition of lipids recovered after defatting was determined by gas chromatography following conversion to fatty acid methyl esters (FAMEs) according to David et al. [34]. Approximately 100 mg of ethanol-extracted oil from TMLP was weighed into a methylation tube, and 100 µL of internal standard (C23:0, 2% *w*/*v* in n-heptane) was added. Fatty acids were transmethylated by adding 1.0 mL of 0.5 M methanolic NaOH and heating in boiling water for 5 min. After cooling, 1.5 mL of 20% boron trifluoride was added; this was followed by a second heating step of 5 min. After cooling to room temperature, 5 mL of saturated NaCl solution and 2.5 mL of n-heptane containing 0.01% BHT were added. The samples were vigorously shaken for 30 s and allowed to undergo phase separation. The upper organic phase was collected and transferred to GC vials. FAMEs were analyzed using a gas chromatography system (HP-7890A, Agilent Technologies, CA, USA). Finally, the concentrations of individual fatty acids present in the samples were determined by internal calibration, using C23:0 FAME as the internal standard, together with theoretical correction factors (TCFs) and empirical correction factors (ECFs) based on peak areas relative to the internal standard. Analyses were performed in duplicate, and the results were expressed as mg g^−1^ total fatty acids. Details of the internal calibration and quantification procedures for fatty acids are provided in File S3.

### 2.6. Statistical Analysis

All statistical analyses were performed using JMP Student Edition 2018 (SAS Institute Inc., Cary, NC, USA). The effects of the factors (probiotics and UST) were analyzed within the framework of the general linear model by fitting the obtained data to a factorial linear regression model (Equation (3)) [35].

(3) $$Y=\beta_{0}+{\beta_{1}D}_{1}+{\beta_{2}D}_{2}+{\beta_{3}D}_{3}+\beta_{4}t+\beta_{5}D_{1}t+\beta_{6}D_{2}t+\beta_{7}D_{3}t+\varepsilon$$

where *Y* is the observed response and *t* represents time, while the feed groups are represented as dummy variables (n − 1). *D*_1_, *D*_2_ and *D*_3_ correspond to BC, BV and PP, respectively, with Std. taken as the reference group. *β*_0_ represents the intercept; *β*_1_, *β*_2_, and *β*_3_ represent the regression coefficients for the feed groups; and β4 represents the regression coefficient for time. Furthermore, *β*_5_, *β*_6_, and *β*_7_ represent the regression coefficients for the interaction terms, and ε represents the random error (residual) term of the model, which is assumed to be normally distributed with a mean of zero and constant variance. Model significance was assessed using analysis of variance (ANOVA). Statistical significance was declared at *p* < 0.05.

## 3. Results and Discussion

### 3.1. Protein Yield

The protein yield data are presented in Table 1. Statistical analysis (Table S4) showed that protein yield was significantly affected by both input factors, as well as their interaction, and that the yield varied across sonication times (*p* < 0.05). Among the probiotic treatment groups, BV showed the highest protein yield, whereas BC showed the lowest (*p* < 0.05). The Std. group did not differ significantly from the PP group. In addition, protein yield generally decreased with increasing sonication time from 0 to 25 min, except in the BV group, which showed an increase in yield with increasing sonication time.

The results of this study differ from those reported by Zhang et al. [24] but are consistent with the findings of Zhang et al. [31], who applied a similar extraction protocol to minimally processed larvae. Working with wet larvae may facilitate disruption of hydrated cell membranes, which could reduce the additional benefit of ultrasound treatment compared with the use of dried powder, where cell-wall disruption and mass transfer may be enhanced by UST application [36]. Ultrasound treatment has been reported to enhance extraction efficiency up to a plateau as sonication time increases; however, prolonged exposure may reduce protein yield. This may be attributed to the dual effects of ultrasound, which can promote protein unfolding and dispersion and enhance diffusion, but may also promote protein re-aggregation and reduce solubility, ultimately lowering protein recovery [37]. Moreover, prolonged sonication may increase heat accumulation and cavitation-related chemical effects, which could further contribute to the reduction in protein yield [37]. The findings of this study contrast with those of previous studies in which defatting was performed before protein extraction from insects [28,38,39,40] to improve extraction yield. In particular, Choi et al. [40] reported that combining defatting with sonication improved protein yield.

Apart from the effect of UST, differences in protein yield among the probiotic treatment groups may plausibly be associated with shifts in protein deposition, gut microbial composition, and/or endogenous digestive enzyme activity, which could alter the composition of the starting material before extraction. This possibility is supported by the present findings, in which the BV group showed an improved yield compared with the control group, whereas the BC group showed the lowest yield. Such physiological changes may affect feed conversion and nitrogen assimilation, potentially resulting in differences among dietary treatment groups even when downstream ultrasound treatment does not improve extraction efficiency [41,42,43,44].

In particular, the lower performance of the BC group could potentially be associated with greater protein hydrolysis, which may facilitate protein unfolding. During sonication, exposure of hydrophobic residues may promote protein re-aggregation, thereby reducing protein recovery during extraction [44]. In addition, a more acidic microenvironment in the BC group may have shifted proteins toward their isoelectric region, resulting in reduced solubility, while UST may have accelerated collisions between unfolded proteins and promoted aggregation, thereby reducing recovery [45]. Likewise, longer ultrasound treatment does not necessarily result in higher protein yields, because ultrasound-induced structural changes can affect protein solubility and extractability [46]. The role of lipids and chitin–protein associations should also not be overlooked. The substantial lipid content may hinder protein separation through emulsion formation and lipid–protein hydrophobic interactions, potentially resulting in less-soluble protein–lipid complexes compared with free proteins [38].

Apart from UST, the differences in yield as per probiotics could be linked to plausibly shifted protein deposition, gut microbial composition, and/or endogenous digestive enzyme activity changing the “starting material” before extraction. That is obvious in this study, where BV showed improved yield relative to the control group and the BC group was associated with the smallest effect. Such activities can alter feed conversion and nitrogen assimilation, which can manifest as differences among diet groups even if downstream ultrasound does not help extraction [41,42,43,44]. Particularly for BC strains, the poor performance could be attributed to the more hydrolyzed proteins that could unfold more readily, and with sonication the protein exposes hydrophobic residues and promotes reaggregation, resulting in low recovery of protein from extraction [44]. Also, the more acidic micro-environment in the BC group could have moved proteins to an iso-electric region and caused reduced solubility, where UST application could accelerate collision of unfolded proteins with low precipitation [45]. Likewise, longer ultrasound times do not always equate to higher yields, because structural changes affect solubility and extractability [46]. Another significant role, that of lipids and chitin–protein associations, should not be ignored. The substantial quantity of lipids could hinder protein separation by the formation of emulsions binding proteins, creating lipid–protein hydrophobic interactions which are less soluble than free proteins [38].

### 3.2. Protein Composition and Gel Electrophoresis

#### 3.2.1. Proximate Composition

The data and statistical analysis for dry matter (DM), total ash contents of the final protein extracts, and the fat content of TMLP before defatting are presented in Table 1 and Table S4, respectively. No significant effects (*p* > 0.05) of either input factor or of their interaction were observed for dry matter content. However, UST significantly affected (*p* < 0.05) the total ash content among the treatments. In this study, UST was applied during protein extraction without heating, and all treatment combinations underwent identical processing steps, which may explain the absence of significant differences in DM content [47].

Likewise, total ash content in insect meals depends on the feed provided during rearing, whereas probiotic supplementation has not been reported to significantly contribute to mineral content [48,49]. In the present study, total ash content decreased with increasing UST duration. Since minerals are often associated with insoluble structures such as chitin and salts present in the hemolymph, increased UST duration may improve protein purity by facilitating the removal of non-protein components during extraction, thereby reducing their content in the final protein extracts [50].

In contrast, differences were observed in the fat contents of the non-defatted protein extracts. The control groups had the lowest fat content, whereas co-extraction of lipids was observed in the sonicated samples. Moreover, fat content increased with increasing sonication time. No significant differences (*p* > 0.05) in fat content were detected among the probiotic treatment groups.

Previous studies on aqueous protein extraction from other insect species have similarly reported lipid co-extraction, which may result from the formation of stable emulsions during acoustic cavitation. Acoustic cavitation can promote lipid dispersion in the aqueous phase and entrapment within protein matrices [51,52]. The presence of lipids may induce protein unfolding, exposing hydrophobic regions that favor lipid–protein interactions. This phenomenon may be enhanced by prolonged UST and the associated increase in temperature, which may further promote protein unfolding and lipid binding [4,51]. Although this study did not demonstrate a significant effect of probiotics on fat content, the results indicate that prolonged UST may enhance lipid co-extraction.

#### 3.2.2. Amino Acid Composition

The amino acid composition data are presented in Table 2. Statistical analysis of the amino acid data (Table S5) demonstrated that probiotic supplementation had a greater influence on amino acid composition than UST, which affected only a limited number of amino acids. Furthermore, significant interaction effects between probiotic supplementation and ultrasound duration were observed for specific amino acids, suggesting that the response to ultrasound treatment depended on the probiotic treatment group.

The most notable effects of probiotic supplementation were observed for threonine (THR) and histidine (HIS), which were significantly affected by probiotics, UST, and their interaction (*p* < 0.05). Both amino acids appeared to be responsive to structural and compositional changes associated with UST application. Furthermore, methionine (MET) and lysine (LYS) were significantly affected by probiotic supplementation, whereas phenylalanine (PHE), isoleucine (ILE), and valine (VAL) showed no significant variation across the treatments or ultrasound durations, indicating relative stability.

Among the non-essential amino acids (NEAAs), probiotic supplementation significantly affected glutamic acid (GLU), glycine (GLY), serine (SER), tyrosine (TYR), and proline (PRO) (*p* < 0.05). Likewise, UST significantly affected GLY, SER, and GLU (*p* < 0.05), while significant interaction effects were observed for SER, GLY, and TYR, suggesting a time-dependent effect of UST. The Std. group showed noticeably higher levels of several NEAAs, including GLU, SER, and GLY, whereas the probiotic treatment groups showed lower concentrations. This reduction may be associated with structural modifications, protein degradation, or solubilization effects induced by processing [53].

The improvement in amino acid availability in probiotic-fed larvae is consistent with the reported capacity of *Bacillus* spp. and *P. pentosaceus* to produce extracellular proteases and colonize the gut, thereby potentially enhancing protein hydrolysis and amino acid absorption [54,55,56,57,58,59]. Similar increases in protein and amino acid levels following probiotic supplementation have been reported in mealworms and other biological systems [14]. The selective effects observed for essential amino acids, particularly methionine (MET) and lysine (LYS), may be associated with protease and peptidase activities, which can alter amino acid profiles [41].

In contrast, the levels of some amino acids, including methionine, threonine, and serine, remained comparatively low. This may be related to their susceptibility to oxidation or degradation during processing, particularly under physical treatments such as sonication [37]. Ultrasound application also showed a time-dependent effect. The pronounced responses observed for THR and HIS suggest that these amino acids may be particularly sensitive to ultrasound-associated structural changes. Controlled acoustic cavitation may enhance mass transfer and protein release, whereas excessive ultrasound exposure can generate free radicals, localized heating, and shear forces that may promote peptide bond cleavage and amino acid oxidation [37,60,61].

The higher levels of hydrophobic amino acids following short-duration UST are consistent with previous reports that ultrasound can induce protein unfolding and preferential cleavage at hydrophobic regions, potentially increasing the release or detectability of these residues [23]. In contrast, the stability observed for other amino acids may indicate greater resistance to structural changes and a lower susceptibility to denaturation or enzymatic degradation [62]. Overall, the results demonstrated that probiotic supplementation was the primary factor influencing amino acid composition, whereas ultrasound processing exerted selective effects depending on amino acid composition and treatment duration.

#### 3.2.3. SDS-PAGE

**Figure 1:**
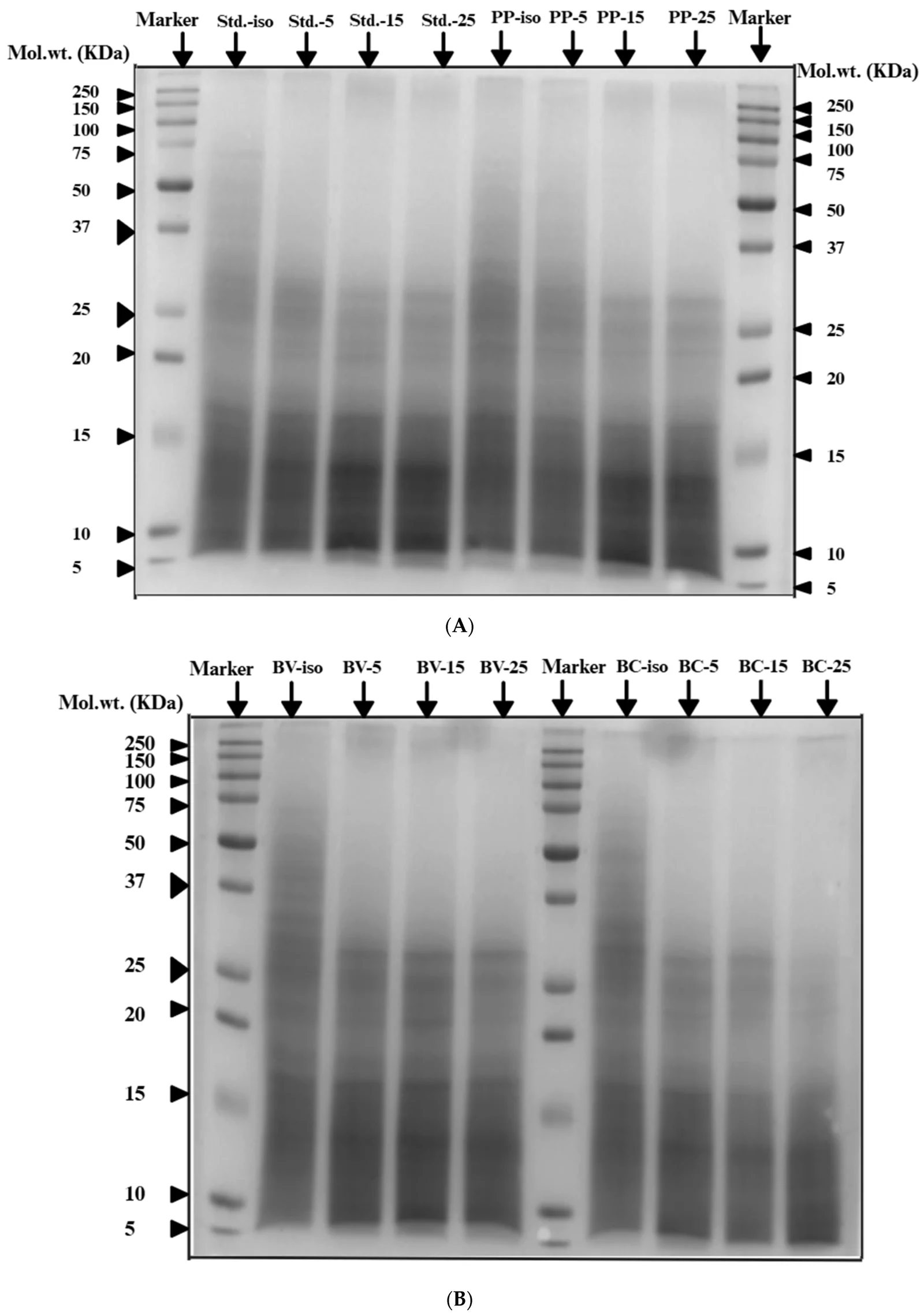
SDS-PAGE profiles of proteins extracted from yellow mealworm larvae subjected to different dietary and ultrasound treatment conditions. (**A**) Standard diet and *Pediococcus pentosaceus*-supplemented diet: Standard-Iso (control), Standard-5, Standard-15, Standard-25, PP-Iso (control), PP-5, PP-15, and PP-25. (**B**) *Bacillus velezensis*- and *Bacillus coagulans*-supplemented diets: BV-Iso (control), BV-5, BV-15, BV-25, BC-Iso (control), BC-5, BC-15, and BC-25. UST, ultrasound treatment; Iso, isoelectric precipitation control.

The SDS–PAGE profiles of TMLP are presented in Figure 1A,B and show clear variations in band intensity and distribution across the different dietary treatments and sonication durations. In both gels (A and B), protein bands were observed across a wide molecular-weight range, with prominent regions around approximately 15, 20, and 25 kDa. A pronounced band at approximately 14 kDa may be associated with antifreeze and hemolymph proteins [63]. The protein bands ranging from 14 to 32 kDa are consistent with *T. molitor* cuticular proteins, while the band around 23 kDa may be attributed to chymotrypsin-like proteinase [64,65]. The bands detected between 37 and 50 kDa could be associated with enzymatic and muscle-related proteins, including melanization-inhibiting protein, α-amylase, and actin-related proteins [1,66].

The protein samples extracted without sonication showed bands between 50 and 75 kDa. These bands may correspond to tubulin α-1 chain (50.6 kDa), putative myosin tail protein (60.1 kDa), calcium-transporting ATPase (72.9 kDa), and tropomyosin 1 (approximately 75 kDa) [66]. In the sonicated samples, changes in protein-band intensity and distribution became more apparent with increasing sonication time from 5 to 25 min. Low-intensity bands at approximately 15 kDa and below were evident across all treatments, particularly in the BC-25 group. This pattern may indicate increased protein fragmentation and formation of smaller peptides following the application of acoustic energy [53].

In addition, UST may expose hydrophobic regions through protein unfolding, thereby promoting protein–protein interactions and aggregation, which could hinder protein migration during electrophoresis [61]. The samples subjected to 5 min of UST showed comparatively fewer changes, suggesting that a threshold level of ultrasonic energy may be required before substantial structural disruption occurs [61]. Furthermore, visual differences among the dietary treatment groups suggest that probiotic supplementation may influence protein expression and metabolism. Such changes could contribute to differences in the protein profiles observed among the treatment groups.

### 3.3. Determination of Free Sulfhydryl (SH) Groups

The data obtained from the measurement of free sulfhydryl (SH) groups and the corresponding statistical analysis are presented in Table 3 and Table S6, respectively. The analysis showed that UST was the only factor that significantly affected free SH content (*p* < 0.05), whereas neither probiotic supplementation nor the interaction between probiotics and UST had a significant effect (*p* > 0.05) on the SH contents of TMLP samples. SH content increased progressively with increasing UST duration across all dietary treatments. The extracts obtained without sonication (control extraction; isoelectric precipitation) showed the lowest SH values (approximately 22–26 µmol), whereas 25 min of UST resulted in the highest values (approximately 38–42 µmol), which were significantly higher than those observed at shorter treatment durations. Thus, the increase in SH content was primarily associated with ultrasound treatment and was independent of probiotic supplementation.

SH content reflects changes in protein conformation, including protein unfolding and alterations in tertiary structure [22,67]. Ultrasound treatment may increase the concentration of free SH groups by disrupting disulfide (S–S) bonds and exposing buried cysteine residues through acoustic cavitation [25]. The gradual increase in SH content up to 25 min of UST suggests progressive structural modification and increased molecular flexibility, although this interpretation should be considered cautiously because protein oxidation was not directly measured in this study. Similar trends were reported by Li et al. [67], who associated increases in SH content with reduced particle size and exposure of internal thiol groups resulting from shear and turbulence. The absence of a decrease in SH content with increasing sonication duration suggests that substantial oxidation of free thiol groups did not occur under the conditions investigated. Although minor numerical differences were observed among the probiotic treatment groups, these differences were not statistically significant, confirming that UST, rather than probiotic supplementation, was the primary factor associated with changes in SH content in these protein powders.

### 3.4. Color Measurement

The color characteristics of TMLP samples and the corresponding statistical analysis are presented in Table 3 and Table S4, respectively. Lightness (L*) ranged from 51.81 (PP-5) to 60.41 (BV-25). The BV group generally produced lighter protein powders, particularly after 25 min of UST (60.42 ± 2.84), whereas the PP group showed lower L* values, particularly after 5 min (51.81 ± 2.12) and 15 min (52.14 ± 2.18) of UST. However, statistical analysis showed no significant effects of probiotic supplementation, UST, or their interaction on the color parameters, except for the yellow–blue coordinate (b*), which was significantly affected by probiotic supplementation (*p* < 0.05).

The total color difference (ΔE) ranged from 1.44 ± 1.13 to 3.54 ± 1.00, with no significant effects of probiotic supplementation, UST, or their interaction (*p* > 0.05), indicating that the overall color differences remained within a similar range [31]. Similarly, the browning index (BI) ranged from 32.09 ± 2.40 to 39.24 ± 2.67, with no significant treatment effects (*p* > 0.05). However, the significant lack of fit observed for the model suggests that some variability in the color data was not adequately explained by the fitted model. Therefore, potential contributions from factors such as protein composition, lipid oxidation, and Maillard reactions should be interpreted cautiously [68,69,70,71].

### 3.5. Hydrophobicity

The data obtained from the measurement of surface hydrophobicity (H_0_) of TMLP samples and the corresponding statistical analysis are presented in Table 3 and Table S6, respectively. The analysis showed that both input factors, as well as their interaction, significantly affected surface hydrophobicity (*p* < 0.05). Among these factors, UST exhibited a stronger effect than either probiotic supplementation or its interaction with UST. Among the probiotic treatment groups, BV showed the highest surface hydrophobicity, whereas BC, PP, and the standard diet showed comparatively lower values. With the increase in UST duration from 0 to 25 min, surface hydrophobicity generally decreased across the samples. A clear interaction effect was observed: the BV group exhibited high hydrophobicity in the absence of ultrasound and after a short UST duration (5 min), but hydrophobicity decreased with increasing sonication time. In contrast, the BC and PP groups showed relatively minor changes following UST, indicating a weaker response to ultrasound treatment.

The observed differences may be associated with probiotic-induced structural rearrangements in proteins that could alter the accessibility of hydrophobic residues [22]. Studies have reported that probiotic symbionts can regulate host protein systems, including stress-responsive and metabolic proteins, through modulation of gene expression [72]. Such interactions may influence protein folding and stability, ultimately affecting the exposure of hydrophobic residues on the protein surface [22,67]. When hydrophobic regions remain buried within the protein structure, ANS may have limited access to these regions, resulting in lower measured surface hydrophobicity because the measurement depends on probe accessibility rather than the total hydrophobic content of the protein [73].

The values obtained in this study were comparable to those reported in previous studies [1,23,62,73]. The higher hydrophobicity observed in the BV group could be associated with greater exposure of non-polar amino acid residues, potentially resulting from partial protein unfolding [25]. In addition to probiotic supplementation, UST duration and its interaction with probiotic treatment significantly affected the hydrophobicity of TMLP (*p* < 0.05), indicating that probiotic supplementation may modulate the response of proteins to different ultrasound conditions. Surface hydrophobicity decreased with increasing sonication time, which contrasts with the findings of Huang et al. [74]. Extended sonication may promote the reassociation of hydrophobic domains and the formation of small aggregates, thereby reducing the measured surface hydrophobicity [18,22]. Similar trends have been reported by Zhang et al. [25]. The significant interaction effect further suggests that probiotic feeding may alter the susceptibility of proteins to ultrasound-induced structural changes rather than directly determining their surface hydrophobicity.

### 3.6. Intrinsic Fluorescence

**Figure 2:**
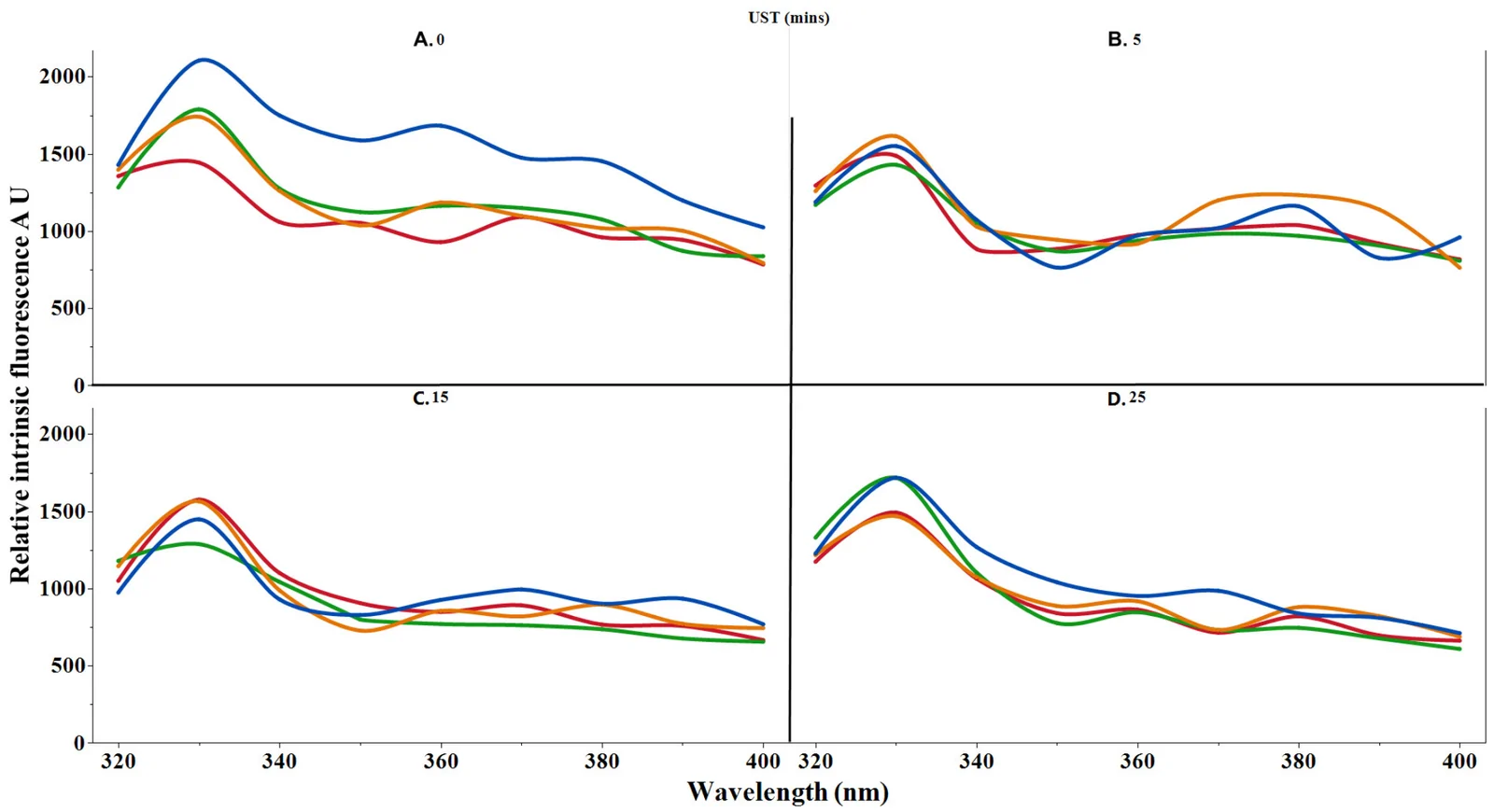
Relative fluorescence intensity (RFI, a u) of protein samples extracted from four probiotic groups as indicated above, with respective color legends as indicated. Standard control diet, (Std., blue), *Pediococcus pentosaceus* (PP, orange), *Bacillus velezensis* (BV, green) and *Bacillus coagulans* (BC, red) at four different ultrasound pretreatment times: (**A**) (0 min), (**B**) (5 min), (**C**) (15 min) and (**D**) (25 min), respectively. The X-axis represents the wavelength (nm) while Y-axis represents the intrinsic fluorescence emitted by the protein samples, expressed as relative intrinsic absorbance.

The intrinsic fluorescence spectra of TMLP under different probiotic treatments and ultrasound pretreatment (UST) conditions, together with the corresponding statistical analysis, are presented in Figure 2, Table 3, and Table S6, respectively. The statistical analysis showed that neither the input factors (probiotic supplementation and UST) nor their interaction had a significant effect (*p* > 0.05) on the relative fluorescence intensity (RFI) of TMLP. Intrinsic fluorescence is widely used to monitor protein unfolding through changes in the microenvironment of aromatic amino acid residues, particularly tryptophan, which is highly sensitive to its surrounding environment [75]. Although fluorescence intensity showed some numerical variation across UST durations, these differences were not statistically significant. Under the control (0 min) condition, all samples exhibited similar fluorescence intensities, indicating comparable baseline protein conformations. Following 5 and 15 min of UST, slight increases in fluorescence intensity were observed in some probiotic treatment groups, particularly BC and BV, whereas PP and the standard diet showed relatively smaller changes. At 25 min, fluorescence intensity appeared to decrease in some groups, particularly BV and BC. Because these changes were not statistically significant, they should be interpreted as numerical variation rather than evidence of treatment-induced structural modifications.

### 3.7. Protein Solubility

The data obtained from the protein solubility experiments are presented in Table 3, and the corresponding statistical analysis is provided in Table S6. Statistical analysis of protein solubility at each pH (2, 5, 7, and 11) showed that UST was the only input factor that had a statistically significant effect (*p* < 0.05) on solubility at pH 2 and 7, whereas probiotic supplementation had no significant effect on protein solubility. At pH 2 and 7, protein solubility ranged from approximately 50.6 to 83.2%, and UST significantly affected solubility, with increasing UST duration generally resulting in decreased solubility (*p* < 0.05), except for the PP group at pH 2 and the BC group at pH 7 and 11. Protein solubility depends on pH, ionic conditions, and mechanical forces [76,77,78]. Acoustic cavitation and the resulting structural disruption may promote protein aggregation rather than dispersion, as well as expose hydrophobic residues, thereby favoring aggregation under acidic conditions [20,25]. In addition, the decrease in solubility with increasing sonication time may be associated with protein denaturation, structural changes, unfolding, and subsequent reaggregation [24].

Overall, the solubility data demonstrated a strong dependence on pH, with higher solubility under acidic and alkaline conditions and very low solubility at pH 5, indicating a non-linear relationship. High solubility under strongly acidic conditions may reflect a strong net positive charge on the proteins and enhanced protein–water interactions away from the isoelectric point [77], consistent with previous reports for mealworm proteins [63]. At pH 5, solubility was minimal (0.00–6.62%), with no significant treatment effects (*p* > 0.05), indicating that the proteins were close to their isoelectric region, where the net charge approaches zero and protein–protein interactions become dominant [77]. Although the data showed a tendency toward decreased solubility with longer UST durations (15–25 min), this effect was not statistically significant (*p* > 0.05). The decrease was more apparent in the Bacillus-fed groups, potentially due to reaggregation following protein unfolding [78,79,80].

Under alkaline conditions, protein deprotonation results in increased negative charges, enhancing electrostatic repulsion and protein solubility [81]. Probiotic supplementation may also subtly modify protein composition and hydration behavior [79,80]. At pH 11, solubility was highest (approximately 78.1–91.7%) across all samples, with only small differences among the treatment groups. The high solubility under alkaline conditions may be attributed to increased deprotonation and negative charge, which enhance electrostatic repulsion between protein molecules and promote solubility [22,67,81,82].

### 3.8. Particle Size and ζ-Potential Measurement

**Figure 3:**
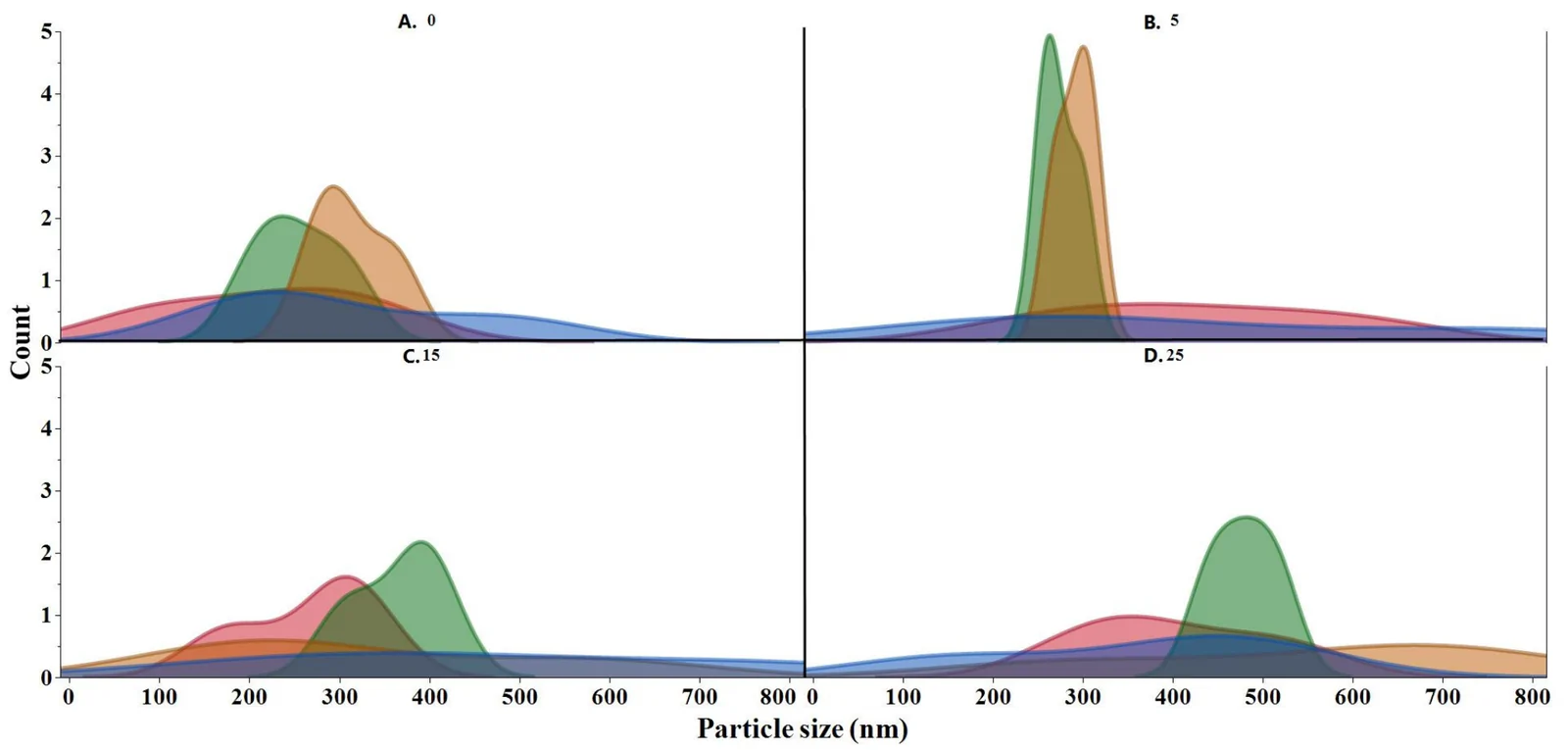
Graphs showing particle size expressed as hydrodynamic diameter (nm) for all probiotic groups, with respective colors as indicated: Standard (Std., blue), *Pediococcus Pentosaceus* (PP, orange), *Bacillus velezensis* (BV, green), and *Bacillus coagulans* (BC, red). The X-axis represents the particle size of TML protein powder while the Y-axis is the frequency distribution (expressed in counts from 0 to 5). The particle distribution for all the feed groups is grouped as per sonication time (0 (**A**), 5 (**B**), 15 (**C**) and 25 (**D**) min, respectively, from top left, top right, bottom left and bottom right, respectively. Particle size was expressed as D (3,2), and D (4,3), where D (3,2) represents volume–surface mean diameter and D (4,3) represents the volume–mean diameter. The respective colored shaded areas represent the frequency distribution of particles within each treatment group; the position of each distribution indicates the corresponding particle-size range, while the height represents the relative frequency of particles detected. Overlapping colored areas indicate similar particle-size distributions between treatment groups.

**Figure 4:**
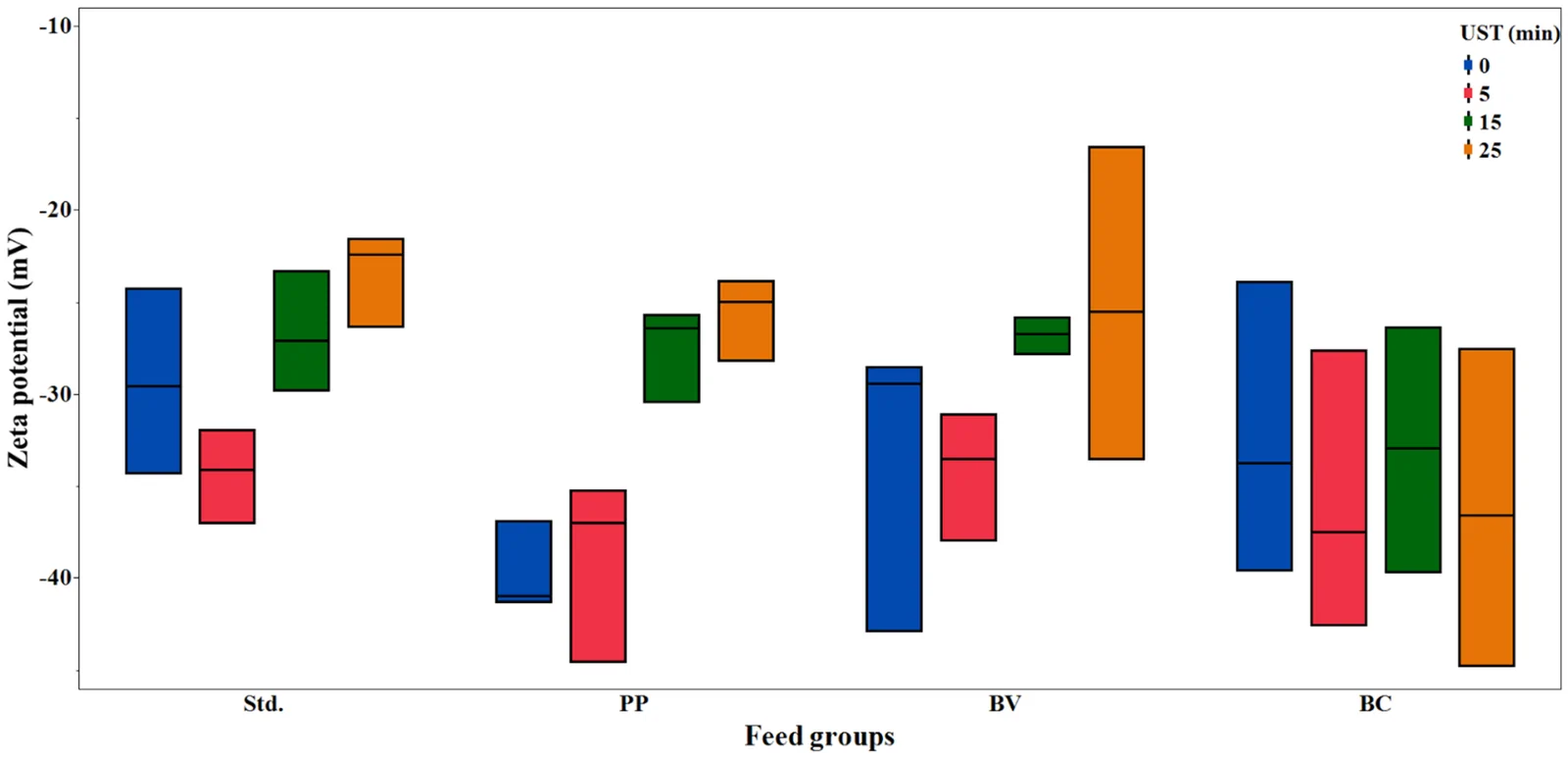
Box plot graphs showing zeta potential (mV) for all feed groups: Standard (STd.), *Pediococcus Pentosaceus* (PP), *Bacillus velezensis* (BV) and *Bacillus coagulans* (BC). The X-axis represents feed groups against zeta potential (mV). The box plot is based on quantile, in which the lower bottom is the first quantile, the center line is the median, and upper areas denote the third quartile. Each sonication period is represented with colors, as indicated: 0 (blue), 5 (red), 15 (green) and 25 (orange) min, respectively (legends inside the graph are also provided).

The particle size and ζ-potential of protein extracts from larvae reared on different probiotic diets (Std., PP, BV, and BC) are presented in Figure 3 and Figure 4, respectively, and the corresponding statistical analyses are provided in Table S6. Although the mean hydrodynamic diameter appeared to increase numerically at longer sonication durations (15–25 min) in some treatment groups (Std., PP, and BV), statistical analysis revealed no significant effects (*p* > 0.05) of diet group, ultrasound treatment (UST), or their interaction on protein particle size. Therefore, these numerical differences should only be considered descriptive and do not provide evidence that UST altered the particle size of the protein extracts under the experimental conditions investigated in this study. Previous studies have reported that prolonged ultrasound treatment may promote protein reaggregation following the initial disruption of protein aggregates [83].

All samples exhibited negative ζ-potential values (approximately −23 to −40 mV), indicating electrostatic stabilization. Unlike particle size, ζ-potential was significantly affected by both probiotic supplementation and UST, but not by their interaction. Moreover, increasing UST duration generally corresponded to a decrease in the magnitude of the negative charge on the protein molecules, except in the BC group (Table S6). The generally higher negative charge observed in the probiotic-fed groups may be associated with microbial enzymatic activity, such as protease and amylase activity, which may modify protein composition and expose acidic residues [84]. A similar mechanism has been reported in other probiotic-treated protein systems [85]. Overall, probiotic supplementation and ultrasound-assisted extraction did not significantly alter particle size, whereas both factors significantly affected the surface charge of the protein molecules.

### 3.9. Fourier Transform Infrared Spectroscopy (FTIR)

The FTIR Amide I deconvolution data are presented in Table 4. The TMLP extracts analyzed in this study were dominated by β-sheet structures (approximately 41–47%), followed by β-turns (approximately 20–31%), with lower contributions from α-helices (approximately 12–22%) and random coils (approximately 12–15%). A β-sheet-rich structural profile has been widely reported for insect proteins, particularly *Hermetia illucens*, in which FTIR analyses commonly identify β-sheets as the major conformational component, often accounting for approximately 40–50% of the secondary structure [86]. This predominance has been linked to the intrinsic composition of insect proteins, including cuticle-associated and chitin-interacting proteins that are structurally enriched in extended β-type conformations [86].

In addition, protein secondary-structure distributions measured by FTIR are known to vary with processing history, including drying, defatting, pH exposure, and extraction conditions, owing to partial unfolding, refolding, or aggregation phenomena that can redistribute α-helical and β-turn structures without necessarily altering the overall dominance of β-sheet structures [87]. The magnitude of the differences observed in the present dataset remained within the ranges reported for insect protein materials and processed insect flours, supporting the structural plausibility of the measured distributions and their consistency with the literature. Differences in α-helix and β-turn contributions among the TMLP preparations may be associated with biological variability among the larvae and post-harvest processing, both of which are known to alter insect protein composition and conformation [1,88].

Studies conducted on black soldier fly (BSF) have shown that rearing substrate and diet can strongly influence larval nutrient composition, including protein and amino acid content, which may indirectly affect the conformational distribution of extracted proteins [89]. In addition, BSF performance and biomass composition can be influenced by microbial supplementation during rearing. For example, supplementation with *Bacillus* strains has been shown to alter gut microbial communities and increase larval protein content under certain conditions, supporting the possibility that different feeding strategies, including probiotic supplementation, may contribute to differences in protein structure measured after extraction [10,41,90]. Finally, several studies have demonstrated that processing and extraction methods, including defatting, alkaline extraction/precipitation, enzymatic hydrolysis, and heat exposure, can induce measurable structural changes in TML proteins, including changes in the Amide I and Amide II regions observed by FTIR. These findings are consistent with the possibility that the balance between α-helix and β-turn structures may vary among preparations without substantially altering the overall tendency of TML proteins to adopt β-sheet-rich structures [88].

### 3.10. Differential Scanning Calorimetry (DSC)

The thermal properties of the TMLP extracts are presented in Table 5, including glass transition temperature (Tg), denaturation temperature (Td), melting temperature (Tm), and their corresponding enthalpies. The statistical analysis of these thermal properties is presented in Table S7. Both glass transition temperature (Tg) and glass transition enthalpy (ΔHg) were significantly affected by probiotic supplementation and UST (*p* < 0.05). In contrast, neither probiotic supplementation nor UST had a significant effect (*p* > 0.05) on denaturation and melting temperatures. However, UST significantly affected their corresponding enthalpies (ΔHd and ΔHm) (*p* < 0.05). Furthermore, increasing sonication duration resulted in a decrease in ΔHd, whereas ΔHm increased across all treatment groups.

The Tg values ranged from 40.90 to 48.04 °C, with the lowest value observed for the control extraction from the standard-diet group and the highest value observed for the BC-25 treatment. In general, the treated samples exhibited higher Tg values than the standard isothermal condition; however, statistical analysis did not demonstrate a significant difference for this trend. An increase in Tg is typically associated with reduced molecular mobility and enhanced intermolecular interactions, such as protein–protein associations and cross-linking, or with decreased plasticization by water or low-molecular-weight solutes. These results may suggest that the applied processing conditions promoted structural rearrangements leading to a more rigid amorphous phase [25]. The probiotic treatment groups showed comparably elevated Tg values, suggesting a similar degree of molecular mobility restriction among these samples. This behavior is consistent with previous studies showing that processing-induced aggregation and network formation can shift Tg to higher temperatures [91].

Despite the differences in Tg, ΔHg values remained low (0.21–0.89) and did not differ significantly among the samples. This suggests that the energetic requirement associated with the glass transition was relatively unaffected by the input factors. Small variations in ΔHg are often attributed to subtle differences in amorphous-phase organization rather than large-scale structural transformations [92].

Denaturation temperatures were narrowly distributed between 87.15 and 90.19 °C, with no significant differences among the samples. This indicates that the intrinsic thermal stability of the protein structures was largely preserved across the treatments. Td is primarily associated with the unfolding of ordered protein domains, and the relatively stable Td values suggest that the processing conditions did not substantially disrupt the thermal stability of the dominant protein fractions [93]. In contrast, denaturation enthalpy (ΔHd) showed a decreasing trend across the treatments, from approximately 1.75 to 0.30–0.61, with increasing UST duration. Lower ΔHd values indicate that less energy was required to induce denaturation, which may be interpreted as evidence of partial pre-denaturation, aggregation, or conformational rearrangements occurring before DSC analysis. Processing treatments that promote protein–protein interactions or partial unfolding can reduce the cooperativity and energy requirement of thermal denaturation [94,95].

Melting temperatures (Tm) ranged from 122.02 to 133.91 °C and did not differ significantly among the samples, indicating the comparable thermal stability of crystalline or highly ordered domains. Although ΔHm values varied numerically (0.10–1.32), the absence of statistically significant differences suggests some heterogeneity but overall, a similar extent of ordered structures. Variations in melting enthalpy are commonly associated with differences in crystallinity or ordered aggregate content; however, the present results indicate that these differences were not systematic across the probiotic treatment groups. Overall, the data demonstrate that the applied processing conditions primarily affected amorphous-phase mobility and the energetic aspects of protein denaturation, while leaving the characteristic denaturation and melting temperatures largely unchanged. This pattern suggests that processing influenced supramolecular organization and intermolecular interactions to a greater extent than the intrinsic thermal stability of the proteins [95].

## 4. Fatty Acid Analysis

The fatty acid data and corresponding statistical analyses are presented in Table 6 and Tables S7 and S8, respectively. The analysis showed that probiotic supplementation was the dominant factor affecting fatty acid composition, whereas UST exerted selective effects and significantly interacted with probiotic treatment. Among the individual fatty acids, probiotic supplementation had significant effects (*p* < 0.05). For example, 14:0, 15:0, and 16:0 showed significant probiotic-dependent differences in concentration (*p* < 0.05), with the PP treatment yielding the highest concentrations, whereas BC resulted in significantly lower values. Similar trends were observed for unsaturated fatty acids, such as 16:1 and 18:2, indicating a systematic increase in fatty acid concentrations under PP treatment. These findings suggest that probiotic supplementation may modulate enzymatic activity, lipid biosynthesis, and host lipid metabolism, as reported in previous studies [14,96]. Total saturated fatty acids (SFA), monounsaturated fatty acids (MUFA), and polyunsaturated fatty acids (PUFA) were all significantly affected by probiotic supplementation (*p* < 0.05), with PP consistently producing the highest levels and BC the lowest. The higher SFA levels observed in some probiotic groups may reflect altered lipid storage and metabolism during larval development [14] and may also provide greater stability against rapid oxidation. Oleic acid (C18:1 n-9) was the dominant MUFA and increased with probiotic supplementation and longer sonication durations. Moreover, the higher PUFA content in the PP group suggests that probiotic activity may promote lipid bioconversion processes, such as desaturation and elongation of fatty acids [97].

In contrast to probiotic supplementation, significant effects of UST were observed for selected fatty acids, including 15:0, 16:0, and 16:3, as well as for the omega-6/omega-3 ratio (*p* < 0.05), but UST did not significantly affect the total SFA, MUFA, or PUFA contents. Longer sonication durations generally decreased fatty acid concentrations across the treatment groups, except for PP. In addition, increasing UST duration was associated with increased concentrations of 18:3(n-4) and decreased concentrations of 18:4(n-3). These findings suggest that physical processing may influence mass transfer, cell disruption, and the release of intracellular compounds without necessarily altering total lipid content [98]. Ultrasound-assisted extraction may also promote the release of endogenous antioxidants and phospholipids, which can contribute to emulsion stability [23]. Acoustic cavitation can disrupt cell membranes and lipoprotein complexes, potentially improving lipid recovery but also requiring process optimization to minimize pro-oxidative conditions [23].

Importantly, a significant interaction between probiotic supplementation and UST (*p* < 0.05) was observed for several fatty acids and lipid classes, including SFA, MUFA, and PUFA. This interaction suggests that the effect of sonication may depend on the probiotic treatment, with UST increasing fatty acid concentrations under the PP treatment while reducing or having minimal effects under the BC treatment. Similar synergistic effects have been reported in other studies, particularly with respect to PUFA concentrations [99]. In addition, the omega-6/omega-3 ratio was significantly influenced by both probiotic supplementation and UST, with increasing sonication duration decreasing the ratio (*p* < 0.05). This ratio represents the relative proportion of omega-6 fatty acids (e.g., linoleic acid and arachidonic acid) to omega-3 fatty acids (e.g., α-linolenic acid, EPA, and DHA) and is considered favorable when approximately 1:1 to 4:1 and less favorable when greater than 10:1 [100]. The reduction in this ratio under certain UST conditions may indicate a potential improvement in the nutritional profile, as lower omega-6/omega-3 ratios have generally been associated with more favorable health outcomes [100]. Overall, these findings indicate that probiotic supplementation was the dominant factor influencing fatty acid composition, whereas ultrasound acted primarily as a modulating factor.

## 5. Conclusions

This study systematically evaluated how probiotic supplementation during larval rearing and ultrasound-assisted extraction influence the nutritional, structural, and functional properties of yellow mealworm (*T. molitor*) protein extracts. The study investigated aqueous protein extraction from minimally processed frozen larvae using ultrasound-assisted alkaline protein extraction. With the increase in UST duration from 0 to 25 min, only the BV group showed a comparable improvement in protein yield relative to the control extraction method. Increasing UST duration from 0 to 25 min also resulted in increased free SH content, decreased protein solubility, altered thermal properties, lower surface hydrophobicity, and a decrease in surface negative charge, indicating structural alterations that may affect protein functionality. In contrast, UST did not significantly affect particle size or intrinsic fluorescence intensity under the experimental conditions investigated.

Probiotic supplementation had a limited effect on protein yield but primarily modified the nutritional composition, particularly the amino acid and fatty acid profiles, and influenced selected physicochemical properties, including surface charge, surface hydrophobicity, and color. In contrast, ultrasound treatment primarily affected protein extraction efficiency, structural characteristics, and functional properties, while exerting selective effects on the amino acid and fatty acid profiles.

Overall, the findings indicate that both biological (probiotic feeding) and physical (ultrasound-assisted extraction) interventions act through complementary mechanisms to tailor insect protein quality rather than simply increasing extraction yield. Because this study used wet larvae directly, in the frozen state, for protein extraction without defatting, the relatively low protein yield highlights the potential importance of incorporating drying and defatting steps into conventional extraction procedures. These findings provide insights into the development of minimally processed mealworm protein ingredients and support the optimization of sustainable extraction strategies for future food applications. Further studies should focus on process scale-up and evaluation of the resulting protein ingredients in food applications to facilitate their potential industrial implementation.

## Figures and Tables

**Table 1:** Protein yield and proximate composition of protein extracted from Tenebrio molitor (TMLP) larvae fed different diets (Std., PP, BV, and BC) and subjected to different ultrasound pretreatment durations (0, 5, 15, and 25 min).

Feed Groups	Ultrasound, UST (Min)	Protein Final Yield (%)	Dry Matter of TMLP (%)	Total Ash of TMLP (%)	Fat % Prior Defatting of TMLP
**Standard control diet (Std.)**	0	30.48 ± 1.42	91.48 ± 4.32	8.65 ± 0.79	11.05 ± 1.06
***Pediococcus pentosaceus*** **(PP)**	0	33.88 ± 4.19	93.83 ± 2.40	7.60 ± 0.79	8.47 ± 0.82
***Bacillus velezensis*** **(BV)**	0	33.88 ± 3.63	93.53 ± 5.27	5.21 ± 0.13	11.14 ± 0.45
***Bacillus coagulans*** **(BC)**	0	29.56 ± 1.53	91.03 ± 2.78	5.73 ± 3.27	7.44 ± 0.82
**Standard control diet (Std.)**	5	29.07 ± 0.47	89.23 ± 2.41	6.91 ± 1.12	40.57 ± 1.50
***Pediococcus pentosaceus*** **(PP)**	5	27.44 ± 4.56	89.00 ± 0.99	7.66 ± 1.24	26.06 ± 3.61
***Bacillus velezensis*** **(BV)**	5	30.35 ± 3.07	92.77 ± 1.44	6.44 ± 2.45	39.43 ± 0.86
***Bacillus coagulans*** **(BC)**	5	24.05 ± 3.97	91.04 ± 3.31	5.91 ± 1.62	24.86 ± 3.48
**Standard control diet (Std.)**	15	26.24 ± 2.55	89.85 ± 2.27	5.19 ± 2.36	45.39 ± 0.84
***Pediococcus pentosaceus*** **(PP)**	15	24.54 ± 1.1	92.85 ± 2.85	4.53 ± 0.72	39.10 ± 1.21
***Bacillus velezensis*** **(BV)**	15	32.02 ± 1.28	94.55 ± 2.47	6.94 ± 0.88	35.56 ± 2.32
***Bacillus coagulans*** **(BC)**	15	21.80 ± 2.17	93.34 ± 0.24	7.23 ± 1.07	38.65 ± 3.37
**Standard control diet (Std.)**	25	27.35 ± 1.7	90.81 ± 1.36	5.25 ± 1.84	36.51 ± 3.25
***Pediococcus pentosaceus*** **(PP)**	25	26.25 ± 0.78	89.95 ± 1.14	3.64 ± 1.31	44.72 ± 2.04
***Bacillus velezensis*** **(BV)**	25	33.56 ± 1.48	87.04 ± 1.91	3.61 ± 1.27	42.30 ± 4.25
***Bacillus coagulans*** **(BC)**	25	17.57 ± 2.51	91.74 ± 1.38	5.07 ± 2.06	32.00 ± 2.76

**Table 2:** Amino acid profiles of larvae fed different diets (Std., PP, BV, and BC) and subjected to ultrasound pretreatment for 0, 5, 15, and 25 min. Amino acid concentrations are expressed as mg g^−1^ of total protein on a dry-weight basis.

	Std.-iso	PP-Iso	BV-iso	BC-iso	Std.-5	PP-5	BV-5	BC-5	Std.-15	PP-15	BV-15	BC-15	Std.-25	**PP-25**	**BV-25**	**BC-25**
**Essential amino acids (mg/g)**																
**Phe**	39.20 ± 4.87	37.39 ± 10.88	43.19 ± 1.90	37.34 ± 3.53	47.25 ± 2.03	43.69 ± 2.87	69.73 ± 34.42	48.32 ± 6.20	54.13 ± 5.33	49.72 ± 4.97	44.30 ± 1.31	43.45 ± 1.52	48.07 ± 1.91	50.22 ± 2.26	41.03 ± 2.33	42.54 ± 1.61
**Leu**	59.62 ± 2.87	48.02 ± 2.25	43.90 ± 2.09	44.90 ± 1.15	57.53 ± 4.38	44.44 ± 3.83	64.85 ± 25.57	52.30 ± 8.49	54.50 ± 3.78	44.63 ± 5.89	46.16 ± 0.14	46.29 ± 0.50	49.89 ± 1.59	47.44 ± 0.98	42.23 ± 1.14	42.82 ± 1.26
**Ile**	40.83 ± 1.82	35.90 ± 2.03	31.92 ± 1.79	32.76 ± 0.87	40.62 ± 2.76	33.63 ± 3.80	53.81 ± 26.29	39.90 ± 6.81	40.29 ± 3.21	36.48 ± 2.04	35.09 ± 0.12	35.09 ± 0.25	39.56 ± 0.96	36.65 ± 0.41	32.91 ± 0.79	33.03 ± 0.53
**Met**	20.77 ± 1.01	15.63 ± 0.69	12.57 ± 0.29	11.45 ± 1.27	21.43 ± 0.74	13.83 ± 1.40	12.01 ± 2.73	15.03 ± 3.06	20.66 ± 1.01	13.41 ± 1.08	12.82 ± 1.00	12.49 ± 0.12	19.35 ± 0.38	13.95 ± 0.17	10.67 ± 2.63	12.21 ± 0.33
**Val**	44.50 ± 2.53	44.77 ± 2.43	42.74 ± 1.59	42.53 ± 1.50	43.91 ± 3.17	42.44 ± 3.65	44.31 ± 0.54	49.44 ± 7.53	43.99 ± 2.62	43.62 ± 4.55	45.00 ± 0.77	44.75 ± 0.86	44.52 ± 0.91	45.47 ± 0.70	42.93 ± 1.92	42.70 ± 0.54
**Thr**	32.77 ± 1.19	21.55 ± 1.04	10.46 ± 0.64	10.15 ± 0.28	35.40 ± 3.87	17.97 ± 3.37	10.06 ± 0.81	12.72 ± 2.38	30.42 ± 2.21	10.75 ± 1.03	10.48 ± 0.29	10.01 ± 0.10	25.23 ± 1.51	10.92 ± 0.10	9.95 ± 0.80	9.75 ± 0.25
**His**	25.96 ± 0.78	16.51 ± 1.75	8.31 ± 0.37	8.48 ± 0.51	25.10 ± 1.92	13.03 ± 2.08	8.46 ± 0.60	9.69 ± 2.10	26.72 ± 1.84	10.64 ± 1.07	8.87 ± 0.18	7.65 ± 0.11	25.82 ± 1.35	8.67 ± 0.24	8.12 ± 1.20	6.83 ± 0.18
**Lys**	63.28 ± 5.95	91.44 ± 17.07	88.79 ± 7.80	86.51 ± 5.30	54.52 ± 4.52	93.31 ± 13.04	82.08 ± 12.72	104.11 ± 27.41	63.68 ± 9.56	86.91 ± 5.51	84.43 ± 1.16	77.02 ± 1.74	70.68 ± 2.24	84.19 ± 4.40	76.03 ± 3.54	67.50 ± 0.66
**Non-essential amino acids (mg/g)**																
**Tyr**	47.97 ± 1.18	33.41 ± 1.39	31.77 ± 1.56	31.13 ± 1.46	47.18 ± 2.66	31.49 ± 3.03	36.42 ± 2.93	38.21 ± 6.88	42.96 ± 3.01	34.46 ± 2.84	36.29 ± 1.43	32.59 ± 0.83	39.23 ± 1.85	36.54 ± 0.97	33.04 ± 1.80	30.65 ± 0.93
**Pro**	29.86 ± 1.18	27.77 ± 0.90	24.65 ± 0.70	25.21 ± 0.52	29.75 ± 1.92	25.31 ± 1.82	26.23 ± 0.66	27.93 ± 3.06	29.62 ± 1.53	24.03 ± 4.06	27.11 ± 0.64	25.85 ± 0.34	28.93 ± 1.04	27.25 ± 0.56	25.58 ± 1.23	25.10 ± 0.49
**Ala**	32.06 ± 1.31	28.49 ± 0.22	21.12 ± 1.31	21.80 ± 0.41	31.35 ± 2.55	25.53 ± 2.79	20.29 ± 2.20	24.78 ± 4.69	29.37 ± 1.79	21.43 ± 1.92	21.03 ± 0.13	20.94 ± 0.20	27.78 ± 0.84	21.73 ± 0.44	19.78 ± 0.82	19.98 ± 0.27
**Gly**	27.47 ± 1.64	23.00 ± 2.67	12.69 ± 0.69	13.23 ± 0.21	28.62 ± 2.68	17.82 ± 3.76	12.89 ± 1.20	16.24 ± 3.33	27.43 ± 1.59	14.16 ± 1.10	14.30 ± 0.18	13.72 ± 0.13	27.07 ± 0.56	14.20 ± 0.26	13.66 ± 1.02	12.77 ± 0.07
**Ser**	30.54 ± 0.86	15.68 ± 4.50	7.19 ± 0.21	7.43 ± 0.09	34.87 ± 4.13	10.67 ± 3.18	7.16 ± 0.81	8.22 ± 1.63	25.93 ± 1.34	7.82 ± 0.11	7.32 ± 0.26	7.23 ± 0.15	22.48 ± 1.62	7.58 ± 0.30	7.11 ± 0.26	7.00 ± 0.11
**Arg**	50.87 ± 3.12	59.09 ± 2.22	55.75 ± 1.69	58.00 ± 1.48	44.81 ± 4.03	54.68 ± 4.00	55.16 ± 5.00	62.59 ± 9.89	50.86 ± 5.02	57.47 ± 0.96	57.92 ± 1.25	54.10 ± 0.21	58.11 ± 3.04	57.08 ± 1.25	53.15 ± 4.78	51.42 ± 1.34
**Glu**	122.02 ± 14.07	99.63 ± 6.12	96.00 ± 5.63	97.71 ± 1.84	147.70 ± 44.11	91.03 ± 10.16	88.93 ± 11.35	102.13 ± 18.73	95.63 ± 3.41	88.18 ± 6.46	89.95 ± 1.10	84.41 ± 1.20	89.92 ± 1.82	92.35 ± 2.29	85.02 ± 2.63	79.45 ± 0.91

**Table 3:** Surface hydrophobicity, free sulfhydryl (SH) content, intrinsic fluorescence, color parameters, and solubility of TMLP extracted from larvae fed different diets (Std., PP, BV, and BC) and subjected to ultrasound pretreatment (UST) for 0, 5, 15, and 25 min. The control treatment (0 min) represents protein extraction by isoelectric precipitation without ultrasound pretreatment.

	Std.-iso	PP-Iso	BV-iso	BC-iso	Std.-5	PP-5	BV-5	BC-5	Std.-15	PP-15	BV-15	BC-15	Std.25	PP-25	BV-25	BC-25
**Hydrophobicity (H^0^)**	318.33 ± 91.35	435.73 ± 168.34	832.63 ± 159.03	396.89 ± 116.92	310.22 ± 36.11	281.13 ± 87.50	904.63 ± 433.14	230.67 ± 65.85	209.07 ± 5.37	160.02 ± 25.79	174.55 ± 68.38	213.98 ± 60.93	164.52 ± 42.88	148.97 ± 31.61	177.54 ± 20.95	141.92 ± 13.65
**Free SH contents (µmol)**	22.36 ± 1.84	23.85 ± 1.90	26.32 ± 1.52	23.11 ± 2.33	33.38 ± 8.14	28.56 ± 5.92	26.09 ± 3.26	26.89 ± 7.94	27.66 ± 3.98	31.32 ± 0.56	32.01 ± 0.86	31.50 ± 2.35	38.25 ± 1.76	35.31 ± 5.18	42.44 ± 7.68	39.35 ± 5.53
**λ _max_ at 330 nm**	2105.48 ± 10.40	1741.36 ± 14.11	1788.89 ± 12.31	1443.92 ± 295.23	1550.56 ± 23.04	1614.64 ± 15.94	1429.17 ± 158.51	1487.53 ± 174.82	1448.01 ± 0.36	1564.99 ± 18.08	1287.40 ± 268.91	1575.44 ± 6.10	1717.52 ± 15.26	1468.93 ± 99.87	1717.20 ± 14.86	1492.37 ± 161.74
**Colour measurement**																
Lightness (L*)	56.73 ± 1.59	57.13 ± 1.85	60.03 ± 1.48	58.69 ± 0.91	54.20 ± 4.98	51.81 ± 2.12	55.49 ± 1.28	54.21 ± 3.02	53.31 ± 1.58	52.14 ± 2.18	56.04 ± 4.62	54.21 ± 3.02	58.50 ± 0.95	56.29 ± 0.92	60.42 ± 2.84	57.51 ± 1.30
a*	0.39 ± 0.53	1.78 ± 0.69	0.51 ± 0.12	0.77 ± 0.35	1.49 ± 0.40	1.28 ± 0.91	1.97 ± 0.38	2.09 ± 0.50	2.25 ± 0.10	1.82 ± 0.23	2.30 ± 0.70	2.09 ± 0.50	1.23 ± 0.01	1.71 ± 0.14	1.07 ± 0.60	0.94 ± 0.21
b*	15.27 ± 1.18	17.58 ± 0.91	16.74 ± 0.59	16.68 ± 0.69	15.98 ± 1.22	14.89 ± 0.84	17.92 ± 0.83	16.79 ± 0.21	16.03 ± 0.44	13.58 ± 1.59	18.12 ± 1.13	16.79 ± 0.21	16.88 ± 0.13	17.50 ± 1.07	18.55 ± 0.21	17.03 ± 0.62
**∆E**		3.03 ± 0.97	2.32 ± 0.88	2.65 ± 1.47		3.42 ± 2.28	2.74 ± 1.36	2.88 ± 1.35		3.54 ± 1.55	2.76 ± 1.44	2.88 ± 1.35		2.71 ± 1.15	3.01 ± 1.39	1.44 ± 1.13
**Browning index (BI)**		38.25 ± 1.66	32.71 ± 1.28	33.63 ± 1.60		35.12 ± 4.39	34.88 ± 2.41	39.24 ± 2.67		32.09 ± 2.40	36.77 ± 2.35	39.24 ± 2.67		38.63 ± 1.79	37.24 ± 2.71	35.49 ± 0.87
**Solubility (%)**																
**pH 2**	83.21 ± 3.94	62.31 ± 10.26	67.09 ± 16.14	60.21 ± 19.16	65.46 ± 7.22	61.03 ± 10.28	72.71 ± 4.02	72.50 ± 10.84	69.01 ± 5.65	65.98 ± 5.35	66.98 ± 4.01	50.63 ± 11.12	56.81 ± 2.32	68.31 ± 1.95	59.96 ± 6.77	51.77 ± 10.27
**pH 5**	5.91 ± 6.01	2.61 ± 4.52	0.01 ± 0.00	0.00 ± 0.00	0.00 ± 0.00	0.00 ± 0.00	0.00 ± 0.00	2.83 ± 4.89	0.00 ± 0.00	0.00 ± 0.00	0.00 ± 0.00	3.16 ± 5.48	0.00 ± 0.00	0.01 ± 0.00	0.00 ± 0.00	6.62 ± 11.45
**pH 7**	29.78 ± 8.42	32.85 ± 3.92	27.07 ± 4.47	27.71 ± 2.21	25.64 ± 3.19	32.22 ± 6.40	29.12 ± 3.32	34.40 ± 4.99	26.86 ± 3.62	25.36 ± 4.86	20.08 ± 3.00	19.93 ± 2.02	19.25 ± 1.27	19.63 ± 2.49	18.44 ± 5.69	26.66 ± 5.85
**pH 11**	91.72 ± 3.58	85.33 ± 4.47	82.57 ± 3.14	80.09 ± 2.64	81.94 ± 2.76	82.08 ± 2.68	83.80 ± 4.77	79.63 ± 9.83	82.22 ± 2.41	83.56 ± 4.43	83.14 ± 4.36	85.33 ± 2.24	82.74 ± 2.48	82.97 ± 4.92	78.07 ± 3.16	82.29 ± 2.12

Note: Values are mean ± SD from triplicate measurements.

**Table 4:** Secondary structure composition of proteins extracted from yellow mealworm larvae fed different diets (Std., PP, BV, and BC) and subjected to ultrasound pretreatment (UST) for 0 (Iso), 5, 15, and 25 min.

Sample	β-Sheet (%)	α-Helix (%)	Random Coil (%)	β-Turn (%)
**STD-Iso**	45.70	12.50	13.10	28.60
**STD-5A**	46.80	12.90	12.20	28.10
**STD-15**	44.80	12.30	12.90	30.00
**STD-25**	44.90	22.20	13.00	19.90
**PP-Iso**	45.00	11.90	13.10	30.00
**PP-5**	45.20	22.00	12.70	20.10
**PP-15**	44.10	22.10	12.60	21.20
**PP-25**	46.70	21.20	11.80	20.30
**BV-Iso**	45.20	12.60	13.00	29.20
**BV-5**	44.70	12.20	12.50	30.60
**BV-15**	44.80	22.40	12.50	20.20
**BV-25**	43.90	22.30	12.30	21.60
**BC-Iso**	41.40	14.30	14.60	29.70
**BC-5**	44.80	13.90	14.40	26.90
**BC-15**	43.00	14.00	14.80	28.10
**BC-25**	43.10	14.00	14.80	28.10

**Table 5:** Thermal properties of TMLP extracted from larvae fed different diets (Std., PP, BV, and BC) and subjected to ultrasound pretreatment (UST) for 0 (Iso), 5, 15, and 25 min.

	Std.-iso	PP-Iso	BV-iso	BC-iso	Std.-5	PP-5	BV-5	BC-5	Std.-15	PP-15	BV-15	BC-15	Std.25	PP-25	BV-25	BC-25
**Glass temperature, T_g_ (°C)**	40.90 ± 0.61	42.74 ± 0.23	44.41 ± 0.00	46.00 ± 0.74	45.40 ± 0.60	45.36 ± 0.09	47.23 ± 0.34	47.37 ± 0.20	43.79 ± 0.26	44.78 ± 0.01	45.19 ± 0.02	45.99 ± 0.98	47.39 ± 0.21	47.13 ± 0.62	47.38 ± 0.15	48.04 ± 0.00
**Heat Enthalpy, ∆E_g_**	0.89 ± 0.17	0.48 ± 0.07 ^a^	0.49 ± 0.00	0.21 ± 0.06	0.85 ± 0.00	0.77 ± 0.07	0.81 ± 0.10	0.72 ± 0.03	0.81 ± 0.29	0.45 ± 0.00	0.42 ± 0.02	0.53 ± 0.05	0.73 ± 0.34	0.53 ± 0.01	0.83 ± 0.01	0.63 ± 0.00
**Denaturation temperature, T_d_ (°C)**	88.62 ± 1.22	89.34 ± 0.29	89.65 ± 0.00	89.73 ± 0.16	89.73 ± 0.05	89.29 ± 0.69	89.81 ± 0.03	89.41 ± 0.11	89.81 ± 1.22	89.89 ± 0.06	89.21 ± 0.93	87.15 ± 1.11	89.85 ± 0.20	90.19 ± 0.22	89.74 ± 0.52	89.85 ± 0.00
**Heat Enthalpy, ∆E_d_**	1.75 ± 0.46	1.16 ± 0.35	0.61 ± 0.00	0.42 ± 0.05	0.61 ± 0.04	0.77 ± 0.09	0.88 ± 0.08	1.19 ± 0.06	0.58 ± 0.22	0.52 ± 0.00	0.34 ± 0.03	0.31 ± 0.05	0.42 ± 0.03	0.30 ± 0.2	0.36 ± 0.04	0.36 ± 0.00
**Melting temperature, T_m_ (°C)**	126.19 ± 0.47	125.72 ± 2.9	128.08 ± 0.00	127.12 ± 2.30	124.68 ± 1.56	126.36 ± 2.06	125.23 ± 0.84	127.01 ± 2.71	130.04 ± 3.46	133.91 ± 3.71	128.64 ± 0.42	125.99 ± 3.85	129.91 ± 0.59	129.38 ± 2.49	122.02 ± 1.43	122.56 ± 0.00
**Heat Enthalpy, ∆E_m_**	0.39 ± 0.22	0.33 ± 0.20	0.12 ± 0.00	0.54 ± 0.45	0.54 ± 0.03	0.39 ± 0.18	0.12 ± 0.03	0.10 ± 0.06	0.17 ± 0.07	0.48 ± 0.03	0.44 ± 0.07	1.32 ± 0.87	0.62 ± 0.04	1.23 ± 1.10	0.88 ± 0.03	0.62 ± 0.00

Note: Values are given as means ± SD from duplicate measurements.

**Table 6:** Fatty acid composition of oil extracted from yellow mealworm larvae fed different diets (Std., PP, BV, and BC) and subjected to ultrasound pretreatment (UST) for 0 (Iso), 5, 15, and 25 min. Fatty acid concentrations are expressed as mg/g of total fatty acids. **Note:** Values are presented as mean ± SD from duplicate measurements. The omega-6/omega-3 ratio was calculated from the summed n-6 and n-3 fatty acids.

Fatty Acids (mg/g)	Std.-0	Std.-5	Std.-15	Std.-25	PP-0	PP-5	PP-15	PP-25	BV-0	BV-5	BV-15	BV-25	BC-0	BC-5	BC-15	BC-25
**14:0**	13.91 ± 0.17	15.98 ± 0.38	12.99 ± 0.56	15.4 ± 0.19	16.93 ± 0.11	17.84 ± 0.41	18.0 ± 0.01	20.47 ± 0.75	16.1 ± 0.37	17.74 ± 0.5	17.92 ± 0.72	14.46 ± 0.89	15.31 ± 2.56	13.58 ± 2.04	12.57 ± 0.4	10.56 ± 0.17
**15:0**	1.75 ± 0.03	1.85 ± 0.05	1.43 ± 0.07	1.73 ± 0.02	1.97 ± 0.0	1.95 ± 0.03	1.96 ± 0.04	2.26 ± 0.05	1.81 ± 0.02	1.86 ± 0.04	1.84 ± 0.08	1.52 ± 0.09	1.84 ± 0.26	1.5 ± 0.25	1.36 ± 0.03	1.17 ± 0.02
**16:0**	102.43 ± 0.66	110.21 ± 3.16	75.32 ± 3.13	93.69 ± 1.45	122.83 ± 0.42	122.1 ± 2.8	109.29 ± 0.61	129.45 ± 3.79	113.21 ± 3.8	110.1 ± 3.04	96.69 ± 4.8	91.47 ± 4.21	119.64 ± 9.31	86.56 ± 21.29	73.71 ± 1.83	70.86 ± 1.98
**16:1 (n-7)**	9.58 ± 0.15	11.74 ± 0.07	9.86 ± 0.32	11.76 ± 0.16	11.14 ± 0.14	12.73 ± 0.21	13.51 ± 0.0	15.82 ± 0.39	10.23 ± 0.11	12.52 ± 0.63	13.2 ± 0.4	10.28 ± 0.6	9.95 ± 1.48	10.22 ± 1.23	9.74 ± 0.37	8.4 ± 0.19
**16:3 (n-4)**	2.23 ± 0.45	1.2 ± 0.04	0.82 ± 0.01	1.01 ± 0.03	1.56 ± 0.02	1.21 ± 0.01	1.03 ± 0.07	1.35 ± 0.02	1.42 ± 0.0	1.16 ± 0.11	1.06 ± 0.12	0.86 ± 0.0	1.66 ± 0.11	0.87 ± 0.25	0.66 ± 0.03	0.66 ± 0.02
**17:0**	1.78 ± 0.11	1.58 ± 0.05	1.31 ± 0.06	1.6 ± 0.0	1.62 ± 0.0	1.72 ± 0.04	1.85 ± 0.04	2.17 ± 0.1	0.72 ± 1.01	1.85 ± 0.29	1.86 ± 0.31	1.36 ± 0.04	1.4 ± 0.17	1.51 ± 0.05	1.31 ± 0.08	1.19 ± 0.06
**18:0**	99.52 ± 5.25	128.85 ± 14.6	96.5 ± 1.46	123.41 ± 0.36	75.93 ± 88.61	140.26 ± 15.52	138.45 ± 0.07	162.45 ± 21.91	121.17 ± 11.22	106.56 ± 6.65	127.95 ± 24.05	96.14 ± 6.79	102.05 ± 0.94	97.01 ± 23.31	68.9 ± 3.9	79.9 ± 37.68
**18:1 (n-9)**	158.8 ± 9.17	170.4 ± 20.1	154.08 ± 11.77	172.87 ± 4.71	144.61 ± 6.09	167.82 ± 11.23	178.82 ± 1.69	208.03 ± 11.97	137.94 ± 8.33	180.11 ± 15.5	185.35 ± 17.45	157.3 ± 3.82	171.39 ± 33.82	147.51 ± 10.28	159.18 ± 0.96	126.63 ± 30.3
**18:2 (n-6)**	214.77 ± 3.5	222.18 ± 4.16	184.17 ± 8.98	213.55 ± 3.14	263.47 ± 1.86	264.93 ± 3.89	256.68 ± 1.21	333.12 ± 10.52	214.43 ± 1.65	229.47 ± 7.15	241.23 ± 8.35	178.22 ± 8.37	253.7 ± 20.6	201.2 ± 50.41	170.7 ± 4.94	166.45 ± 3.52
**18:2(n-4)**	0.51 ± 0.01	0.61 ± 0.02	0.51 ± 0.03	0.58 ± 0.01	0.48 ± 0.0	0.52 ± 0.0	0.47 ± 0.0	0.66 ± 0.02	0.42 ± 0.0	0.55 ± 0.01	0.54 ± 0.01	0.4 ± 0.04	0.48 ± 0.06	0.39 ± 0.13	0.0 ± 0.0	0.16 ± 0.22
**18:3 (n-4)**	0.74 ± 0.01	0.56 ± 0.59	0.86 ± 0.83	1.18 ± 0.05	0.32 ± 0.03	0.54 ± 0.15	0.55 ± 0.02	1.17 ± 0.01	0.59 ± 0.03	0.73 ± 0.2	0.37 ± 0.19	0.89 ± 0.04	0.23 ± 0.33	0.9 ± 0.3	1.12 ± 0.09	1.21 ± 0.02
**18:3 (n-3)**	11.81 ± 0.19	13.88 ± 0.09	11.27 ± 0.41	12.81 ± 0.19	15.42 ± 0.05	16.87 ± 0.27	15.19 ± 0.08	21.1 ± 0.73	11.65 ± 0.11	14.31 ± 0.38	14.54 ± 0.72	10.09 ± 0.49	13.53 ± 1.25	11.7 ± 4.31	8.94 ± 0.38	9.69 ± 0.14
**18:4 (n-3)**	0.54 ± 0.02	0.42 ± 0.03	0.36 ± 0.01	0.39 ± 0.03	0.55 ± 0.01	0.0 ± 0.0	0.25 ± 0.35	0.26 ± 0.36	0.23 ± 0.33	0.0 ± 0.0	0.21 ± 0.3	0.0 ± 0.0	0.9 ± 0.0	0.29 ± 0.42	0.0 ± 0.0	0.0 ± 0.0
**20:0**	0.22 ± 0.01	0.2 ± 0.02	0.18 ± 0.01	0.3 ± 0.0	0.21 ± 0.0	0.48 ± 0.32	0.71 ± 0.23	0.26 ± 0.0	0.38 ± 0.03	0.2 ± 0.04	0.33 ± 0.03	0.75 ± 0.03	0.0 ± 0.0	0.47 ± 0.66	1.1 ± 0.24	0.61 ± 0.05
**20:1 (n-9,n-11)**	2.42 ± 0.03	2.48 ± 0.18	1.98 ± 0.1	2.32 ± 0.04	2.71 ± 0.05	2.59 ± 0.03	2.98 ± 0.17	3.03 ± 0.07	2.64 ± 0.05	2.22 ± 0.12	2.45 ± 0.06	2.27 ± 0.12	2.93 ± 0.26	2.36 ± 0.1	2.71 ± 0.23	1.96 ± 0.08
**20:2 (n-6)**	0.75 ± 0.01	0.63 ± 0.02	0.53 ± 0.02	0.6 ± 0.02	0.89 ± 0.0	0.72 ± 0.01	0.72 ± 0.06	0.93 ± 0.03	0.69 ± 0.0	0.62 ± 0.03	0.65 ± 0.03	0.47 ± 0.03	0.5 ± 0.71	0.33 ± 0.46	0.54 ± 0.06	0.46 ± 0.03
**20:3 (n-3)**	0.0 ± 0.0	0.0 ± 0.0	0.0 ± 0.0	0.0 ± 0.0	0.0 ± 0.0	0.0 ± 0.0	0.17 ± 0.25	0.0 ± 0.0	0.12 ± 0.17	0.0 ± 0.0	0.14 ± 0.01	0.29 ± 0.0	0.0 ± 0.0	0.2 ± 0.29	0.44 ± 0.0	0.19 ± 0.01
**20:4 (n-3)**	0.31 ± 0.0	0.0 ± 0.0	0.0 ± 0.0	0.0 ± 0.0	0.0 ± 0.0	0.0 ± 0.0	0.86 ± 1.22	0.2 ± 0.29	1.11 ± 0.02	0.3 ± 0.01	0.58 ± 0.01	1.47 ± 0.1	0.2 ± 0.29	1.09 ± 1.55	2.27 ± 0.07	1.01 ± 0.02
**20:5 (n-3)**	0.24 ± 0.02	0.0 ± 0.0	0.0 ± 0.0	0.0 ± 0.0	0.0 ± 0.0	0.0 ± 0.0	0.79 ± 1.12	0.29 ± 0.06	1.02 ± 0.03	0.25 ± 0.0	0.56 ± 0.03	1.36 ± 0.1	0.18 ± 0.25	1.02 ± 1.44	2.11 ± 0.08	0.93 ± 0.01
**23:0 STD**	21.92 ± 0.17	17.74 ± 0.98	16.21 ± 2.3	17.1 ± 0.13	19.34 ± 0.73	18.27 ± 1.51	18.92 ± 0.25	25.31 ± 1.94	18.31 ± 1.77	19.82 ± 1.7	18.37 ± 1.29	19.3 ± 1.79	27.6 ± 5.23	19.94 ± 1.4	20.31 ± 1.0	19.7 ± 0.38
**SFA, mg g^−1^**	241.55 ± 4.42	276.42 ± 10.01	203.94 ± 4.66	253.25 ± 1.44	238.82 ± 89.86	302.61 ± 16.97	289.18 ± 0.6	342.36 ± 28.53	271.7 ± 12.67	258.13 ± 1.62	264.96 ± 28.64	225.0 ± 13.83	267.84 ± 18.46	220.56 ± 47.69	179.26 ± 5.48	183.99 ± 40.21
**MUFA, mg g^−1^**	170.8 ± 9.05	184.62 ± 19.99	165.91 ± 11.99	186.95 ± 4.91	158.46 ± 5.99	183.13 ± 11.05	195.31 ± 1.51	226.88 ± 11.51	150.82 ± 8.16	194.86 ± 16.25	201.0 ± 16.98	169.85 ± 4.53	184.27 ± 35.57	160.09 ± 11.4	171.64 ± 1.56	136.98 ± 30.03
**PUFA, mg g^−1^**	231.89 ± 4.13	239.48 ± 4.96	198.52 ± 10.31	230.12 ± 3.47	282.69 ± 1.91	284.79 ± 4.31	276.72 ± 3.52	359.09 ± 12.04	231.68 ± 2.33	247.38 ± 7.65	259.88 ± 9.66	194.05 ± 9.09	271.39 ± 21.85	218.0 ± 52.4	186.77 ± 5.65	180.75 ± 3.46
**USFA, mg g^−1^**	402.69 ± 13.18	424.1 ± 24.95	364.44 ± 22.3	417.07 ± 8.38	441.15 ± 7.9	467.92 ± 6.74	472.03 ± 5.04	585.97 ± 0.53	382.5 ± 5.84	442.24 ± 23.9	460.88 ± 7.32	363.9 ± 13.62	455.65 ± 57.42	378.09 ± 63.81	358.41 ± 7.21	317.73 ± 26.57
**Total omega6, mg g^−1^**	215.52 ± 3.51	222.81 ± 4.18	184.7 ± 9.0	214.15 ± 3.16	264.36 ± 1.85	265.65 ± 3.89	257.4 ± 1.15	334.05 ± 10.55	215.12 ± 1.65	230.1 ± 7.18	241.88 ± 8.39	178.69 ± 8.4	254.2 ± 21.31	201.53 ± 50.87	171.24 ± 5.0	166.91 ± 3.55
**Total omega3, mg g^−1^**	12.89 ± 0.15	14.3 ± 0.12	11.63 ± 0.42	13.2 ± 0.22	15.97 ± 0.06	16.87 ± 0.27	17.27 ± 2.32	21.85 ± 1.44	14.14 ± 0.66	14.85 ± 0.36	16.03 ± 0.97	13.21 ± 0.7	14.81 ± 0.71	14.31 ± 1.46	13.75 ± 0.53	11.82 ± 0.09
**Ω-6/Ω-3**	16.73 ± 0.07	15.58 ± 0.16	15.88 ± 0.19	16.22 ± 0.04	16.55 ± 0.05	15.75 ± 0.02	15.04 ± 1.95	15.3 ± 0.53	15.23 ± 0.59	15.49 ± 0.11	15.1 ± 0.39	13.53 ± 0.08	17.15 ± 0.62	13.97 ± 2.13	12.45 ± 0.12	14.12 ± 0.19

## Data Availability

The data presented in this study are available on request from the corresponding author (Assoc. Prof. Federico Casanova).

## Supplementary Materials

The following supporting information can be downloaded at: https://www.mdpi.com/article/10.3390/insects17090919/s1; File S1: Power output calculation for ultrasound application; File S2: Amino acid analysis protocol; File S3: Fatty acid analysis protocol as well as internal standard calibration; Table S4: Data analysis table for protein yield, proximate composition and color measurement data; Table S5: Data analysis table for amino acid data; Table S6: Data analysis table for protein solubility, particle size, zeta potential, intrinsic fluorescence at 330max, protein hydrophobicity and free SH contents data; Table S7: Data analysis table for thermal stability and summation fatty acids; Table S8: Data analysis table for fatty acids.
