# Supplementary material for: Effects of Ultrasound-Assisted Extraction of Biofractions from Yellow Mealworm Larvae Fed a Probiotic-Enriched Diet
Source: Insects. 2026 Sep 2;17(9):919. doi: 10.3390/insects17090919 (PMC13607018; doi:10.3390/insects17090919)
Supplement: Supplementary file 1 [file insects-17-00919-s001.zip › Table S8.pdf]

Table S8. Effects of probiotic groups (Std., PP, BV and BC) and ultrasound (UST) pretreatment on different fatty acids (mg/g) profile of lipid samples. The table shows the effect estimates, interaction, model fit in terms of Rsquare, $RSquare_{Adj}$, Root Mean Square Error (RMSE) and P value for model and lack of fit Symbols along with feed groups and UST represent the regression coefficient used in model equation (3).

Coefficient, Terms	14:0	15:0	16:0	16:1(n-7)	16:3(n-4)	17:0	18:0	18:1(n-9)	18:2 (n-6)	18:2 (n-4)	18:3(n-4)	18:3(n-3)	18:4(n-3)	20:0	20:1(n-9, n-11)	20:2(n-6)	20:3(n-3)	20:4(n-3)	20:5(n-3)
Std., β_0	15.57^{***}	1.72^{***}	100.70^{***}	11.33^{***}	1.14^{***}	1.55^{***}	110.76^{***}	164.33^{***}	224.72^{***}	0.45^{***}	0.77^{***}	13.28^{***}	0.26^{***}	0.41^{***}	2.49^{***}	0.62^{***}	0.10^{***}	0.60^{***}	0.56^{***}
BC, β_1	-0.33	-0.29^{***}	-15.16^{***}	-1.84^{***}	-0.22^{*}	-0.21	-25.14^{**}	-14.89^{*}	-30.72^{**}	-0.21^{***}	0.12	-2.51^{**}	-0.00	0.16	-0.04	-0.16^{*}	0.11^{**}	0.58^{**}	0.53^{**}
BV, β_2	-2.79^{***}	0.01	1.00	0.21	-0.04	-0.09	1.42	1.45	-10.55	0.02	-0.12	-0.73	-0.15^{*}	0.02	-0.10	-0.02^{**}	0.04	0.28	0.25
PP, β_3	0.89^{*}	0.32^{***}	20.36^{***}	2.17^{***}	0.13	0.30^{*}	21.96^{*}	13.38^{*}	57.94^{***}	0.09^{**}	-0.09	4.09^{***}	-0.00	0.00	0.35^{***}	0.19	-0.05	-0.32	-0.27
UST, β_4	2.89^{***}	-0.09^{**}	-10.19^{**}	0.46	-0.32^{***}	0.06	4.50	5.31	-7.87	-0.03	0.28^{**}	-0.16	-0.14^{*}	0.15^{*}	-0.09	-0.02	0.05	0.20	0.20
BC*UST, β_5	-0.54	-0.20^{**}	-11.37^{*}	-1.26^{**}	-0.10	-0.19	-17.97	-22.66^{**}	-32.22^{**}	-0.15^{**}	0.13	-1.82^{*}	-0.25^{*}	0.17	-0.26^{**}	0.05	0.04	0.24	0.21
BV*UST, β_6	-1.85^{**}	-0.04	-1.37	-0.54	0.07	0.14	-12.21	0.79	-8.78	0.01	-0.20	-0.76	0.08	0.05	-0.01	-0.06	0.05	0.06	0.05
PP*UST, β_7	1.93^{**}	0.23^{**}	11.71^{*}	1.68^{**}	0.23	0.20	30.14^{**}	23.68^{**}	39.16^{**}	0.10^{*}	0.09	2.47^{**}	0.08	-0.11	0.30^{**}	0.05	-0.03	0.00	0.02
R^2	0.80	0.79	0.73	0.75	0.59	0.36	0.45	0.50	0.77	0.71	0.50	0.73	0.48	0.37	0.64	0.40	0.44	0.43	0.44
R^2_{adj}	0.75	0.74	0.66	0.68	0.47	0.17	0.29	0.35	0.70	0.63	0.36	0.65	0.33	0.19	0.54	0.22	0.28	0.27	0.28
RMSE	1.28	0.14	10.90	1.07	0.30	0.35	26.81	18.06	23.60	0.10	0.29	1.83	0.23	0.27	0.22	0.19	0.12	0.63	0.58
p-value	0.0001	0.0001	0.0001	0.0001	0.0013	0.106	0.0269	0.0106	0.0001	0.0001	0.009	0.0001	0.0152	0.0843	0.0003	0.0600	0.0279	0.0331	0.0289
Lack of fit p-value	0.0119	0.007	0.0007	0.0001	0.0001	0.040	0.486	0.0892	0.0015	0.0031	0.379	0.0023	0.0890	0.0137	0.0004	0.8823	0.0555	0.0401	0.0406

*, **, *** Indicates significant effect term at p value of 0.05, 0.01, 0.0001 respectively.
