# Supplementary material for: Effects of Ultrasound-Assisted Extraction of Biofractions from Yellow Mealworm Larvae Fed a Probiotic-Enriched Diet
Source: Insects. 2026 Sep 2;17(9):919. doi: 10.3390/insects17090919 (PMC13607018; doi:10.3390/insects17090919)
Supplement: Supplementary file 1 [file insects-17-00919-s001.zip › Table S7.pdf]

Table S7. Effects of probiotic groups (Std., PP, BV and BC) and ultrasound (UST) pretreatment on thermal stability and total fatty acids (mg/g) of TMLP. The table shows the effect estimates, interaction, model fit in terms of Rsquare, $RSquare_{Adj}$, Root Mean Square Error and P value for model and lack of fit. Symbols along with feed groups and UST represent the regression coefficient used in model equation (3).

	**Thermal stability**						**Total Fatty acids (mg/g)**			
**Coefficient, Terms**	T_g(℃)	ΔH_g	T_m(℃)	ΔH_m	T_d (℃)	ΔH_d	Total MUFA	Total PUFA	Total SFA	Ω-6/ Ω-3
Std., β_0	45.71***	0.64***	0.64***	0.64***	0.64***	0.63***	178.16***	242.54***	250.58***	15.18***
BC, β_1	1.08*					−0.08	−16.77**	−32.48***	−41.69**	-0.89*
BV, β_2	0.55	127.10***	127.10***	127.10***	127.10***	−0.08	1.55	-11.02	2.39	-0.43
PP, β_3	-0.53					0.01	15.91*	61.72***	46.77***	0.41
UST, β_4	1.33**	0.55***	0.55***	0.55***	0.55***	−0.35***	5.68	−7.80	−6.41	-0.72**
BC*UST, β_5	-0.84					0.13	−24.20**	-33.81**	-33.91*	-0.60
BV*UST, β_6	-0.67	89.41***	89.41***	89.41***	89.41***	0.10	0.24	-9.47	-13.34	-0.13
PP*UST, β_7	0.41	0.34	0.02	0.26	0.22	−0.05	25.67**	42.21**	47.56**	0.12
R^2	0.63	0.13	0.53	0.02	−0.03	0.49	0.52	0.77	0.62	0.49
R^2_{adj}	0.52	0.22	0.3905	0.53	0.98	0.33	0.39	0.71	0.51	0.35
RMSE	1.35	0.1797	0.7523	0.3905	0.540	0.36	18.84	24.77	33.01	1.08
p-value	0.0009	0.1454	0.4021	0.7523	0.308	0.020	0.006	0.0001	0.0005	0.011
Lack of fit p-value	0.0001	0.34	0.02	0.26	0.22	0.014	0.065	0.001	0.218	0.011

*, **, *** Indicates significant effect term at p value of 0.05, 0.01, 0.0001 respectively.
