# Supplementary material for: Effects of Ultrasound-Assisted Extraction of Biofractions from Yellow Mealworm Larvae Fed a Probiotic-Enriched Diet
Source: Insects. 2026 Sep 2;17(9):919. doi: 10.3390/insects17090919 (PMC13607018; doi:10.3390/insects17090919)
Supplement: Supplementary file 1 [file insects-17-00919-s001.zip › Table S6.pdf]

**Table S6**. Effects of probiotic groups (Std., PP, BV and BC) and ultrasound (UST) pretreatment on protein solubility (%), particle size (nm), zeta potential (mV), intrinsic fluorescence at $330_{max}$, protein hydrophobicity (AU ml mg-1) and free SH contents (µmol) of TMLP. The table shows the effect estimates, interaction, model fit in terms of Rsquare, $RSquare_{Adj}$, Root Mean Square Error (RMSE) and P value for model and lack of fit. Symbols along with feed groups and UST represent the regression coefficient used in model equation (3).

| | Solubility (%) | | | | | | | | |
|---|---|---|---|---|---|---|---|---|---|
| **Coefficient, Terms** | pH 2 | pH 5 | pH7 | pH 11 | Particle size(nm) | Zeta potential(mV) | Intrinsic fluorescence | Hydrophobicity AU ml $mg^{-1}$ | Free SH contents (µmol) |
| Std., $\beta_0$ | 64.14*** | 0.00 | 25.32*** | 82.85*** | 347.93*** | −31.04*** | 1583.08*** | 300.52*** | 31.20*** |
| BC, $\beta_1$ | −6.14* | −0.00 | 1.58 | −0.84 | −15.27 | −3.47* | -80.25 | -65.35 | −0.21 |
| BV, $\beta_2$ | 2.08 | 0.00 | −2.27 | −1.19 | 6.67 | 1.59 | -29.50 | 181.38** | 1.34 |
| PP, $\beta_3$ | 0.61 | 0.00 | 1.12 | 0.56 | 34.09 | −1.04 | 2.14 | -57.66 | −0.91 |
| UST, $\beta_4$ | −4.77* | 0.00 | −4.97*** | −1.12 | 76.69** | 3.88** | -65.07 | -182.73*** | 6.79*** |
| BC*UST, $\beta_5$ | −2.98 | 0.00 | 2.02 | 2.90 | −29.38 | −5.04** | 95.13 | 75.86 | 1.05 |
| BV*UST, $\beta_6$ | 0.24 | −0.00 | −0.21 | −1.17 | 37.78 | 1.02 | 44.11 | -221.51** | 1.49 |
| PP*UST, $\beta_7$ | 8.30* | 0.00 | −2.31 | 0.47 | 36.98 | 4.18 | -57.48 | 46.78 | −1.49 |
| $R^2$ | 0.29 | 0.18 | 0.49 | 0.20 | 0.27 | 0.44 | 0.22 | 0.63 | 0.52 |
| $R^2_{adj}$ | 0.17 | 0.03 | 0.40 | 0.06 | 0.13 | 0.33 | 0.00 | 0.57 | 0.44 |
| RMSE | 11.03 | 0.00 | 4.72 | 4.54 | 113.73 | 5.50 | 229.19 | 174.12 | 5.58 |
| p-value | 0.035 | 0.292 | 0.0001 | 0.218 | 0.094 | 0.002 | 0.468 | 0.0001 | 0.0001 |
| Lack of fit p-value | 0.492 | 0.001 | 0.016 | 0.293 | 0.319 | 0.765 | 0.052 | 0.166 | 0.448 |

*, **, *** Indicates significant effect term at p value of 0.05, 0.01, 0.0001 respectively.
