# Supplementary material for: Effects of Ultrasound-Assisted Extraction of Biofractions from Yellow Mealworm Larvae Fed a Probiotic-Enriched Diet
Source: Insects. 2026 Sep 2;17(9):919. doi: 10.3390/insects17090919 (PMC13607018; doi:10.3390/insects17090919)
Supplement: Supplementary file 1 [file insects-17-00919-s001.zip › Table S5.pdf]

Table S5. Effects of probiotic groups (Std., PP, BV and BC) and ultrasound (UST) pretreatment on both essential and non-essential amino acids (mg/g) of dry protein weight. The table shows the effect estimates, interaction, model fit in terms of Rsquare, $RSquare_{Adj}$, Root Mean Square Error (RMSE) and P value for model and lack of fit. Symbols along with feed groups and UST represent the regression coefficient used in model equation (3).

	Amino acids																
**Coefficient, Terms**	**PHE**	**LEU**	**ILE**	**MET**	**TYR**	**PRO**	**VAL**	**ALA**	**THR**	**GLY**	**SER**	**ARG**	**HIS**	**LYS**	**GLU**	**ASP**	**Total AAs (mg/g)**
Std., β_0	46.34***	49.04***	378.29***	14.82***	36.36***	242.54***	44.22***	24.02***	16.51***	17.96***	13.15***	55.04***	13.56***	79.20***	95.82***	79.22***	649.47***
BC, β_1	−3.34	-2.70	-2.20	-2.06***	-3.36***	−32.48***	0.52	-2.30***	-5.93***	-4.05***	-5.72***	1.02	-5.51***	3.19	-6.00	0.00	−39.37*
BV, β_2	2.56	-0.25	0.76	-2.88***	-1.96*	-11.02	-0.46	-3.51***	-6.29***	-4.51***	-5.96***	0.36	-5.12***	3.11	-6.28	-2.56	−33.95*
PP, β_3	-0.47	-2.90	-1.53	-0.69	-2.18*	61.72***	-0.07	-0.07	-1.77***	-1.07	-3.08***	2.01	-1.71***	9.31**	-3.32	1.17	−7.20
UST, β_4	1.23	-2.99	-1.14	-0.62	-0.97	−7.80	-0.07	-1.91***	-2.71***	-1.18**	-2.34***	-0.22	-1.15***	-4.46	-10.57**	-2.20	−31.57**
BC*UST, β_5	-0.35	0.63	0.05	0.35	-0.56	-33.81**	-1.02	0.32	1.96*	0.39	1.95*	-4.31**	0.02	-9.32***	-0.56	-4.01	−14.81
BV*UST, β_6	-7.72	-1.93	-2.62	-0.10	1.16	-9.47	0.12	1.45	2.56**	1.79*	2.33***	-0.58	1.1.1*	-0.61	6.18	0.04	3.79
PP*UST, β_7	4.98	3.05	2.05	-0,05	2.97*	42.21**	0.75	-1.50*	-2.86***	-2.94***	-1.36	0.02	-2.50***	0.11	7.54	3.17	13.42
**0.77**	0.13	0.22	0.08	0.80	0.71	0.40	0.03	0.79	0.94	0.89	0.93	0.37	0.96	0.51	0.46	0.18	0.49
R^2_{adj}	-0.01	0.09	-006	0.77	0.66	0.29	-0.13	0.75	0.93	0.87	0.92	0.26	0.96	0.42	0.36	0.04	0.41
RMSE	12.43	9.31	9.11	1.82	4.36	2.09	3.54	2.27	2.34	2.27	2.58	4.88	1.50	12.43	16.82	6.92	59.65
p-value	0.500	0.139	0.781	0.0001	0.0001	0.0028	0.977	0.0001	0.0001	0.0001	0.0001	0.006	0.0001	0.0001	0.0005	0.272	0.0001
Lack of fit p-value	0.158	0.183	0.155	0.261	0.095	0.180	0.387	0.438	0.028	0.173	0.042	0.258	0.545	0.530	0.298	0.640	0.339

*, **, *** Indicates significant effect term at p value of 0.05, 0.01, 0.0001 respectively.
