# Supplementary material for: Effects of Ultrasound-Assisted Extraction of Biofractions from Yellow Mealworm Larvae Fed a Probiotic-Enriched Diet
Source: Insects. 2026 Sep 2;17(9):919. doi: 10.3390/insects17090919 (PMC13607018; doi:10.3390/insects17090919)
Supplement: Supplementary file 1 [file insects-17-00919-s001.zip › Table S4.pdf]

Table S4. Effects of probiotic groups (Std., PP, BV and BC) and ultrasound (UST) pretreatment on protein yield (%), dry matter and ash (%), fat (%), colour measurement and thermal stability of TMLP. The table shows the effect estimates, interaction, model fit in terms of Rsquare, $RSquare_{Adj}$, Root Mean Square Error and P value for model and lack of fit. Symbols along with feed groups and UST represent the regression coefficient used in model equation (3).

	**Yield**	**Proximate composition**			**Colour measurement**				
**Coefficient, Terms**	Protein yield (%)	Dry matter (%)	Total ash (%)	Extract's Fat (%)	L*	a*	b*	ΔE	Browning index (BI)
Std., β_0	27.75***	91.30 ***	5.85***	31.46***	56.08***	1.49***	16.68***		
BC, β_1	−5.04***	0.54	0.11	−4.55	0.05	-0.02	0.15	-0.18	-0.09
BV, β_2	4.74***	0.39	−0.38	1.81	1.97*	-0.01	1.22**	0.38	1.09
PP, β_3	−0.05	0.01	−0.21	−0.16	-1.72*	0.15	-0.79*	3.34***	37.03***
UST, β_4	−2.53***	−0.76	-1.21**	12.60***	0.37	0.15	0.41	-0.61	0.68
BC*UST, β_5	−2.87*	1.39	1.05	-0.87	-0.54	-0.18	-0.25	-0.67	-0.28
BV*UST, β_6	2.89*	−1.97	0.42	-0.96	0.26	0.02	0.35	0.23	0.90
PP*UST, β_7	−0.89	−0.15	−1.03	4.46	-0.27	-0.07	-0.38		
R^2	0.64	0.15	0.29	0.59	0.18	0.08	0.37	0.10	0.08
R^2_{adj}	0.58	0.01	0.16	0.52	0.03	-0.08	0.26	−0.05	-0.06
RMSE	3.33	3.28	1.98	9.32	3.21	0.74	1.22	2.09	3.86
p-value	0.0001	0.263	0.039	0.0001	0.2944	0.8253	0.0056	0.692	0.559
Lack of fit p-value	0.202	0.400	0.483	0.0001	0.0011	0.0001	0.0001	0.400	0.0002

*, **, *** Indicates significant effect term at p value of 0.05, 0.01, 0.0001 respectively.
