# Supplementary material for: Effects of Ultrasound-Assisted Extraction of Biofractions from Yellow Mealworm Larvae Fed a Probiotic-Enriched Diet
Source: Insects. 2026 Sep 2;17(9):919. doi: 10.3390/insects17090919 (PMC13607018; doi:10.3390/insects17090919)
Supplement: Supplementary file 1 [file insects-17-00919-s001.zip › File S2.pdf]

Bioactive<br><br>Department of Food Technology<br><br>National Food Institute	Analysis Instructions No. 223.01	1st ed. Dec 2023<br>chja/ adms/inho
	# Total amino acid analysis	
Total amino acid composition measured by LCMS without derivatization		

1. Purpose

To determine the amino acid composition in solid and liquid samples.

2. Definition

This method identifies and quantifies the majority of the amino acids in a sample (see Table 1), i.e. both amino acids bound in proteins/peptides and free amino acids (if only free amino acids are to be determined, see Bioactive No. 224 Free amino acids without derivatization).
The amino acid content is expressed as weight per weight (typically milligrams of amino acid per sum (grams) of amino acids or milligrams of amino acid per gram of sample).

Proteins consist of amino acids in a specific sequence (Figure 1). The nutritional quality of proteins can be determined by the amino acid profile. Peptides are smaller proteins (<50 amino acids). The sum of amino acids is also a quantitative measure of protein content (molar weight of water is deducted, since hydrolysis of protein will supply one water molecule for each amino acid, see calculation).

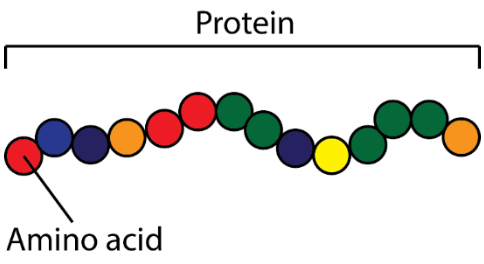

Figure 1. Proteins consist of amino acids linked covalently in chains of more than 50 amino acids

Proteins are the building blocks of cell walls, muscles, etc. Proteins can be quantitatively determined as total N (organic and inorganic N; Dumas) or Kjeldahl N (organic N and ammonia, high concentrations of nitrate can affect ammonia) and then multiply by a factor that converts from nitrogen to protein (see Dumas No. 201). This conversion factor is usually not corrected for species- and seasonal differences, and

the protein content is therefore subject to a high degree of uncertainty for e.g. microalgae and macroalgae (seaweed). However, the method described here on total amino acids overestimates the protein content because the free amino acids are included, but this is thought to be of minor importance.

The proteinogenic ("protein-building") amino acids have an amine and a carboxylic acid group attached to the first (alpha) carbon atom. They are referred to as 2-, alpha or α-amino acids (generic formula most often $H_2NCHRCOOH$, where R denotes a side chain) (Fig. 2)

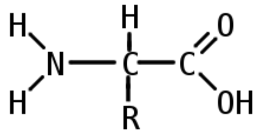

Figure 2. Amino acid

The list below (Table 1) shows all amino acids and which of them we can determine (some decompose or transform during hydrolysis, others lack standards for identification).
Nine amino acids (His, Ile, Leu, Lys, Met, Phe, Thr, Trp and Val) are called essential amino acids, as the human body cannot synthesize them in the amount necessary for normal growth, so they must be ingested with food.
The three non-polar essential amino acids with a "branched" R group are isoleucine, leucine, and valine, and these are interesting since 35% of the essential amino acids in the human muscle have a "branched" chain (branched chained amino acids, BCAA). This is of interest to the fitness and bodybuilding industry as many products specifically describe how much BCAAs are in their product.

Table 1: List of amino acids. The nine essential amino acids are shown in bold**, and the three** branched chain amino acids are italic.MW: molar weight and RET: retention time

	MW + H	Ret time (approx. min)
ARG: Arginine	175	8,3
BE: Serin	106	7,7
HYP: 4-hydroxyproline (not directly incorporated into proteins, but post-translational modification of proline after protein synthesis), HYP is not in std if HYP is desired to be run sepe rat kalibreringkurve	132	5.65
GLY: Glycine	76	7,4
**THR: Threonine**	**120**	**6,6**
ALA: Alanin	90	6,4
PRO: Proline	116	4,9
**MET: Methionine**	**150**	**4,5**
ASP: Aspartic acid. Se note 11.1	134	10,6
SELECTION: Selected	***118***	***5,0***
**HIS: Histidine**	**156**	**8,2**
**LYS: Lysin**	**147**	**8,8**
GLU: Glutamic acid. Note 11.1	148	9,9
**TRP: Tryptophan decomposes completely in this hydrolysis. Not in standard**	**201**	**3,5**
LEU: Leucin	***132***	***3,9***
**PHE: Phenylalanine**	**166**	**3,7**
WITH: Isoleucine	***132***	***4,2***
C-C: Cystin	241	10
TYR: Tyrosine	182	4,9
ASN: Asparagine is converted to ASP in this hydrolysis (see note 11.1)		
CYS: Cysteine decomposes completely in this hydrolysis		
HLY: Hydroxylysine	Not in standard (6.3.7), but standard curves can be made separately	
GLN: Glutamine is converted to glutamic acid (GLU) in this hydrolysis. (See note 11.1)		

3. Scope

This method has previously been applied to meat, brine (herring, shrimp), fish (mackerel, cod, trout), hydrolysates from fish waste, process water, micro- and macroalgae (seaweed) and cheese.

4. Principle

Acid hydrolysis is used when determining total amino acid composition. If only free amino acids are to be measured, the method description "Bioactive No. 224 Free amino acids without derivatization" should be used.

Acid hydrolysis
Proteins and peptides are hydrolyzed using acid and heat to free amino acids. Beware that this method completely breaks down CYS (cysteine) and TRP (tryptophan) and converts ASN (asparagine) and GLN (glutamine) into ASP (aspartic acid) and GLU (glutamic acid), respectively.

Identification and quantification
The amino acids are identified by LC-MS and quantification is done by external standard curves.

5. Appliance

5.1 Scale, 4 decimals.

5.2 Heating oven at 110°C. Microwave Anton Paar 3000 destruction oven (fish samples) see note 11.2

5.3 Agilent LC-MS with column: bioZen 2.6 mym Glycan, 100 x 2.1 mm(00D-4773-AN). Guard ULTRA Cartridges, bioZen Glycan 2.1 mm (AJO-9800) and ULTRA Holder (AJO-9000 (5.11.5), and ion source ESI

5.4 4 mL vials to microwave, (Lab support 41214)

5.5 4 mL vials (f.eks. Fisherman 10571013)

5.6 Screw cap (Lab support 41188) and Teflon inlay (Lab support 41186).

5.7 Pipettes (100 µL, 1 mL, 5 mL)

5.8 1 mL syringes.

5.9 Cellulose acetate 0,22 µm Q-Max RR syringes filter

5.10 pH indicator paper.

5.11 HPLC vials 1,5 mL.

6. Reagents

6.1 12 M HCl for liquid samples, corresponds to 37% HCl.

6.2 6 M HCl for fixed tests. Slowly add 250 ml of 37% HCl (6.1) to 250 ml of distilled water (orange tap).

6.3 1 M KOH, CAS No. 1310-58-3. Weigh 28.05 g and fill up with water to 500 mL.

6.4 0.2 M KOH, CAS No. 1310-58-3. Weigh 5.61 g and fill up with water to 500 mL.

6.5 Formic acid, CAS no. 64-18-6

6.6 Ammonium formiate, CAS no. 540-69-2 (e.g. Aldrich 516961)

6.7 Ion exchange water (orange tap)

6.8 Buffer: 100 mM Ammonium formiate pH 3.1. Weigh 6.3 g ammoniumformiat (6.6) in about 800 mL water. Adjust pH to 3.1 with formic acid (6.5). Fill to 1 L with water

6.9 Acetonitrile, LCMS grade Cas nr 75-05-8

6.10 Mobile Phase A, 10 mM Ammonium formiate in Acetonitrile. 100 mL buffer (6.8) + 900 mL Acetonitrile (6.9)

6.11 Mobile Phase B, 10 mM ammonium formate in water. 100 mL buffer (6.8) + 900 mL of water.

6.12 Amino Acid std: Supelco 79248, 2,5 mM

6.13 Amino Acid std 500 µM: 250 µL (6.12) and 1 mL buffer (6.8)

6.14 Casein house standard (store at -80 °C)

6.15 Seaweed house standarad. (store at -80 °C)

NB: See 13. Safety notes on the labelling of own reagents with danger symbols, H and P phrases.

7. Sample handling

There are no special requirements for handling either solid or liquid samples.

8. Procedure

The analysis is performed as triple determination

Experiments have shown that the finished sample should preferably have a pH of about 3, and this is best achieved by diluting with buffer (6.8).

Each test takes 18 minutes in the LCMS. Do not prepare more tests than can be run on the same day/night.

Acid hydrolysis of the samples

8.1 Weigh sample (approximately 0,5 ml liquid sample (e.g. brine) or 30 mg solid sample (e.g. meat or algae)) in ordinary 4 mL glass (5.5). For hydrolysis in destruction oven use the associated vials (5.4) See note 11.2. Note the exact weight (4 decimal places). Either casein house standard is included (6.14) or seaweed house standard (6.15). Seaweed house standard is used only for seaweed, while casein standard is used for all other samples. Results from these standards are entered into control charts. O:\Bioactive\12. Lipidlab\d. Logbooks plus control charts\Control charts\Control chartsLipid&Oxidation analyzer\control charts2023\Aminoacid test ref. std 2023

8.2 Add HCl: 0.5 ml 12 M (6.1) for liquid samples or 1 ml of 6 M, (6.2) to fixed specimens.

8.3 Cover with nitrogen and close with Teflon inserts and screw caps (5.6)

8.4 Hydrolyze in heating cabinet (or destruction oven). See note 11.2 for method selection.

Heating oven - 110°C for 18 timer:
Place the samples in the oven and check temperature. The samples should be cooled to room temperature before filtration.

Destruction oven - 110 ° C for 60 minutes (500 W; +10 minutes cooling time; method name "amino acids"):
Place the samples in the outer ring of the rotary oven rotor and fill the remaining positions with vials filled with HCl solution. The pre-filled vials with HCl solution are placed permanently near the destruction oven.

8.5 Filter the hydrolysates into 4 ml vials (5.5) through 0.22µm cellulose acetate syringe filters (5.9) using a 1 ml syringe; (5.8).

8.8 Pipettes 100 µL of the filtered hydrolysate into a 4 ml vial (5.5)

8.9 pH adjustment:

Slowly add 1.5 ml of 0.2 M KOH (6,5) to the hydrolysate. Add an additional 1.6 mL buffer (6.8). This gives a dilution factor of 32 (used in the calculation).

If the protein content is low (e.g. brine or liquid extracts, i.e. diluted samples) slowly add 400 µl of 1 M KOH (6.3) so as not to dilute the samples/hydrolysates too much. Add 900 µl buffer (6.8) This gives a dilution factor of 14 (used in the calculation). Remember to correct in the calculation sheet.

8.10 Transfer approx. 1 mL to vials (5.11), and analysis on LCMS

Preparation of calibration curve

Calibration curves can be made at the same time as the samples, but are not made every day. However, always include standard mixture 500 µmol/L (6.13) at the start and end of the sequence. Results are used for control charts (see section 9).

8.11 Std. 500 µmol/L (6.13) injected with 5 different volumes (1, 0.75, 05, 025 and 0.1 µL)

The samples should be analysed on the LCMS on the same day. If this is impossible, they should be set at –18 °C until analysis is possible.

Identification and quantification

8.12 Samples and standards are analysed using method *Aminoacid sim1*. The sequence ends with a blind run by method *aminodown*. (see LC-MS driving directions[1])

Example of sequence building:

Buffer> Standard (6.13) > Sample (s) > Standard (6.13) > Buffer

Method *Aminoacid sim1*

Mobile phase:

A: 10 mM Ammoniumformiate and acetonitrile (6.10)

W: 10 mM Ammonium formate in water (6.11)

Gradient:

0 min	0% B
2 min	5% B
7 min	20% B
8 min	80% B
12 min	80% B
12,1 min	0% B
16 min	0% B

Flow rate: 0,5 mL/min

Column temperature: 40 °C

Injection volume: 1 µL

Method *aminoaciddown*:

Mobile phase: 50% A and 50% B

Flow rate: 0,05 mL/min

[1] .. \.. \.. \b. Equipment manuals\1 HPLC and GC\LC-MS kørselsvejledning.docx

9. Calculation

The instructions on how to make the calibration curve are on the o drive: "O:\Bioactive\12. Lipidlab\b. Equipment manuals\1 HPLC and GC" (click her)
Find the document: "MassHunter Calibration Curve Amino Acids (Quant)" and follow the instructions.
Then find "Amino acid worksheet (excel file)" in the folder: "O:\Bioactive\12. Lipidlab\e. Spreadsheets for analyses (excel sheets for analyses)" (click here).

NOTE: We have two excel files for total amino acids, one for solid samples and one for liquid samples (Amino acid worksheet Total liquid samples New and Amino acid worksheet Total solid samples New).

Unit of result is mg of the individual amino acid/g sample, e.g. mg arginine/g sample (wet weight or dry weight).

Results for the 4 amino acid standards (LEU, PRO, SER and GLU) at a concentration of 500 µM are entered in control sheet O:\Bioactive\12. Lipidlab\d. Logbooks plus control charts\Control chartsLipid&Oxidation analyzer\control charts2023\Amino Acid 2023, together with results from house standards cf. section 8.1. O:\Bioactive\12. Lipidlab\d. Logbooks plus control charts\Control charts\Control chartsLipid&Oxidation analyzer\control charts2023\Aminoacid test ref. std 2023

If the amino acid concentration on the house standards differs more than 14% for all or almost all amino acids, the following should be considered.

1: Check if the calculation has been done correctly

2: Whether the calibration curve is OK

3: If the answer is yes, consider rerunning the tests

Remember: The sum of amino acids is also a quantitative measure of protein. (molar weight of water is deducted, since hydrolysis of protein will supply one water molecule for each amino acid) See example and excel sheet to use in the file "Aminoacid- total_as measure for protein-example" in the folder: O:\Bioactive\12. Lipidlab\e. Amino acid – total as measure for protein–example)

10. Analysis accuracy

Standard deviation is: s is expected to be a maximum of 20%, but further data must be collected before a more accurate value can be provided.

See note 11.3

11. Notes

11.1 During hydrolysis, ASN (asparagine) is converted to ASP (aspartic acid) and GLN (glutamine) to GLU (glutamic acid). Therefore, please note that it is the sum of ASN + ASP and GLN + GLU, respectively, that is determined with this method.

11.2 Microwave oven has been successfully tested on fish samples. Seaweed is not completely hydrolyzed with the microwave. As a general rule, a heating cabinet must be used on test matrices unless it has first been documented that the hydrolysis works satisfactorily when using the microwave oven.

11.3 Comparison of analyses of the 2 house standards for seaweed and casein with results from Eurofins has shown that our values for PHE, LEU, ILE, LYS and MET are slightly below while HIS is slightly above the average values +/- standard deviation of Eurofins. They operate with a standard deviation of 14%. However, this comment is based on a limited number of analyses of house standards.

Results from comparing amino acid composition analyses of the 2 housing standards with Eurofins can be found in the O :\Bioactive\12 folder. Lipidlab\d. Logbooks plus control charts\Control charts\Control chartsLipid&Oxidation analyzer\control charts2023\Aminoacid test ref. std 2023

12. References

Brief method description from Phenomex, which is located in the folder
https://view.highspot.com/viewer/61716e568a59a0c7a63f39c3?iid=5e5823e7a2e3a9095f90318c

Reproducible LC-MS/MS Separation of 38 Amino Acids in HILIC Mode using a bioZen™ Glycan LC Column

13. Safety notices

See workplace instructions for use for HCl (6.1), ammonium formate (6.6) and acetonitrile (6.9). KOH (6.3).

The following reagents must be marked with the symbols of danger, H and P phrases:

Acetonitrile

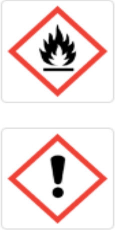

Danger

H225: Highly flammable liquid and vapour.

H302+312+332: Harmful if swallowed, in contact with skin or if inhaled.

H319: Causes serious eye irritation.

P210: Keep away from heat, hot surfaces, sparks, open flames and other ignition sources . No smoking.

P261: Avoid breathing vapours and aerosol

P280: Wear protective gloves/eye protection.

P304+340: IF INHALED: Remove person to fresh air and keep comfortable for breathing.

P305+351+338: IF IN EYES: Rinse cautiously with water for several minutes. Rer contact lenses, if present and easy to do. Continue rinsing.

P301+312: IF SWALLOWED: Call a POISON CENTRE/doctor/ if you feel unwell.

Kaliumhydroxid

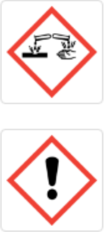

Danger

H290: May be corrosive to metals.

H302: Harmful if swallowed.

H314: Causes severe skin burns and eye damage.

P280: Wear protective gloves/eye protection/face protection.

P301+312: IF SWALLOWED: Call a POISON CENTRE/doctor/ if you feel unwell.

P305+351+338: IF IN EYES: Rinse cautiously with water for several minutes. Rem contact lenses, if present and easy to do. Continue rinsing.

P406: Store in a corrosion-resistant/ container with a resistant inner liner.

Hydrochloric acid 12 m

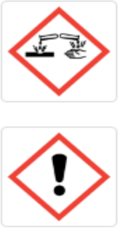

Warning

H290: May be corrosive to metals.

H319: Causes serious eye irritation.

H335: May cause respiratory irritation.

H315: Causes skin irritation.

P280: Wear protective gloves/eye protection.

P302+352: IF ON SKIN: Wash with plenty of water

P305+351+338: IF IN EYES: Rinse cautiously with water for several minutes. F contact lenses, if present and easy to do. Continue rinsing.

P337+313: If eye irritation persists: Get medical advice/attention.

P406: Store in a corrosion-resistant/ container with a resistant inner liner.

14. Waste management

The filtered hydrolysis are emptied into X-acid.
