# Supplementary material for: Effects of Ultrasound-Assisted Extraction of Biofractions from Yellow Mealworm Larvae Fed a Probiotic-Enriched Diet
Source: Insects. 2026 Sep 2;17(9):919. doi: 10.3390/insects17090919 (PMC13607018; doi:10.3390/insects17090919)
Supplement: Supplementary file 1 [file insects-17-00919-s001.zip › File S3.pdf]

<table>
<tr><td rowspan="3">Bioactives

Division of Food Technology
DTU Food</td><td>Analysis Instruction no. 105.03 (11.1)</td><td>1.eds 1985 lbk</td></tr>
<tr><td rowspan="2">GLC-FAME</td><td>2.eds 1995 tv/sd/hm/bj</td></tr>
<tr><td>3.eds march 2018
HASAF/LISBE/INHO/TTTV/
CHJA</td></tr>
<tr><td colspan="3">Gas-Liquid Chromatography of Fatty Acid Methyl Esters</td></tr>
</table>

1. Aim

Determination of the saturated, *cis*-monounsaturated and *cis*-polyunsaturated fatty acids in marine and other oils containing long-chain polyunsaturated fatty acids by capillary GLC in relative (area %), and absolute (weight/weight) values.

2. Definition

This method describes a procedure for the determination of fatty acid methyl esters (FAME's) derived by transesterification or esterification from marine and other fats, oils, and fatty acids by capillary gas chromatography (GLC). Fatty acid methyl esters from C12 to C24 can be separated using this method including saturated fatty acid methyl esters, monounsaturated fatty acid methyl esters, and polyunsaturated fatty acid methyl esters (see 11.2).

3. Field of application

This method determines the content and concentrations of saturated fatty acids (SFA), *cis*-monounsaturated fatty acids (MUFA), and *cis*-polyunsaturated fatty acids (PUFA), including arachidonic acid (ARA), eicosapentaenoic acid (EPA), and docosahexaenoic acid (DHA), in marine oils and other oils such as single cell oils (SCOs) using capillary GLC. This method is not suitable for the analysis of dairy, ruminant fats and oils, or products supplemented with conjugated linoleic acid (CLA) (see 11.3). This method is not applicable for the determination of *trans* fatty acids, *di*-, *tri*- and polymerized fats and oils and microbial oils containing *iso* or branched fatty acids.

4. Principle

Using capillary gas chromatography, FAMEs are separated on a highly polar stationary phase with respect to their chain length, degree of (un)saturation, and geometry and position of the double bonds. Identification of the fatty acids is being done by external standards. Quantification is being done by internal calibration using (C23:0 FAME), and Theoretical correction factors (TCF) and empirical correction factors (ECF). See **Appendix 1** and **Appendix 2.**
TCFs are used to quantitate *all* saturated and monounsaturated fatty acids greater than or equal to 12:0 and polyunsaturated fatty acids (PUFA) of 18 carbons. TCFs are also used for fatty acids, which lack standards, such as 6*c*, 9*c*, 12*c*, 15*c*, 18*c*-21:5 (21:5n-3). ECFs are used for long chain PUFA of 20 carbons or more and three or more double bonds for which standards are readily available.
Very long chain fatty acids (VLC FAs) may be present in single cell oils and certain marine fats and oils. VLC FAs (25:0 and greater) require a longer run time with a pressure flow ramp for elution.
Phthalate esters are also known to be present in marine oils, but their identification is not described in this method.

5. Apparatus

5.1 Gas chromatography system (HP-7890 A, Agilent Technologies, CA, USA).

5.2 Capillary injection system (split mode operated at a split ratio of 1:50). Injection port split liner; 4 mm i.d., 6.3 mm o.d. × 78.5 mm base deactivated precision (Focus) split liner with glass wool (Agilent liner 4 mm ID LPD part nr. 5183-4647) is required for quantification.

5.3 Flame ionization detector, operating at 270-300 °C, amplifier and electronic recorder-integrator.

5.4 GC column DB WAX 127-7012 (10 m x 100 μm x 0.1 μm), from Agilent Technologies (CA, USA) or any other suitable columns.

5.5 Column oven, capable of performing temperature programming at the range 100-270 ±0.1 °C.

5.6 Carrier gas, helium, 99.99% pure or better, is required. Nitrogen is *not* acceptable as a carrier gas for this method.

5.7 Flame gases, nitrogen, hydrogen and air, gas chromatography quality.

5.8 Autosampler system. Agilent technologies.

5.9 Data interpretation-processing software Agilent ChemStation.

6. Reagents

6.1 Reference fatty acid methyl esters (FAME) for control chart **Nu-Check-prep 68D**. Vial with 50 mg is solubilized in 10 ml heptane and divided in portions of 50 µl in inserts to vials, whereafter it is stored in –18 ^{o}C freezer.

6.2 Reference FAME for identification, **Nu-Check-prep GLC 714**

6.3 n- Heptane, (HPLC quality)

7. Handling of sample

Samples shall be prepared in GC vials (1.5 mL), sealed properly and analyzed on the same day. If this is not possible they should be stored in the freezer and be analysed within 1 week. If samples have to be stored for a longer time than 1 week, they should be covered with nitrogen before the vial is sealed and put in the freezer.
The first sample in an analysis batch is always blank (*n*-heptane (6.3)). No peaks other than the solvent should be detected in the chromatogram. The second sample and the last in every analysis batch is always the 68D standard mix (6.1)

8. GLC operating conditions

8.1 Injection port; Injection volume; 0.2 µl (shall be equal to ca. 4-10 µg of total FAME's, see also 9.11), in split mode (1:50). Temperature; 250 °C. Head pressure 51 psi.

8.2 Temperature program (oven), The initial condition is set as 160 °C, followed by, 160–200 °C (rate; 10.6 °C/ min), 200 °C constant for 0.3 min, 200–220 °C (rate; 10.6 °C/min), 220 °C constant for 1 min, 220–240 °C (rate; 10.6 °C/min) and constant at 240 °C for 3.8 min.

8.3 Detector port, Temperature 250°C.

9. Procedure

9.1 Check manually that the injection needle can be moved up and down

Use the following procedure to create, save and run a sequence:

9.2 Select tab: **"Method and Run Control"**

Create a sequence:

9.3 Choose "Sequence". Choose "Sequence Parameters…" Fill in Operator Name and Subdirectory. Choose "Prefix/Counter" and fill in "Prefix" (e.g. yy mm dd). Finish with "OK"

9.4 Choose "Sequence". Choose "Sequence table". Fill in columns: "Vial", "Sample Name", "Method Name" (Find and select the newest method, e.g. FAME 33), "Inj/Vial" and "Inj Volume" (Second last column). Finish with "OK"

Save the sequence:

9.5 Choose "Sequence". Choose "Save Sequence Template As…" Fill in "File name" Finish with "OK"

9.6 Place samples in auto sampler tray, and check the heptane level in the bottles on the autosampler and also in discard the waste. The volume of heptane in the heptane bottle must be over the marked level and the waste bottle must not be full. The order of samples in auto sampler tray should always be:

Heptane (6.3) >STD 68D (6.1) > >Sample(s)……… >> STD 68D (6.1)

Run the sequence:

9.7 Choose "Sequence", "Partial Sequence…", "Select All" "Run Sequence"

Follow the procedure described below for data processing;

9.8 Select tab "**Data Analysis**"

Choose "File", "Load Signal", Select file and finish with "OK"

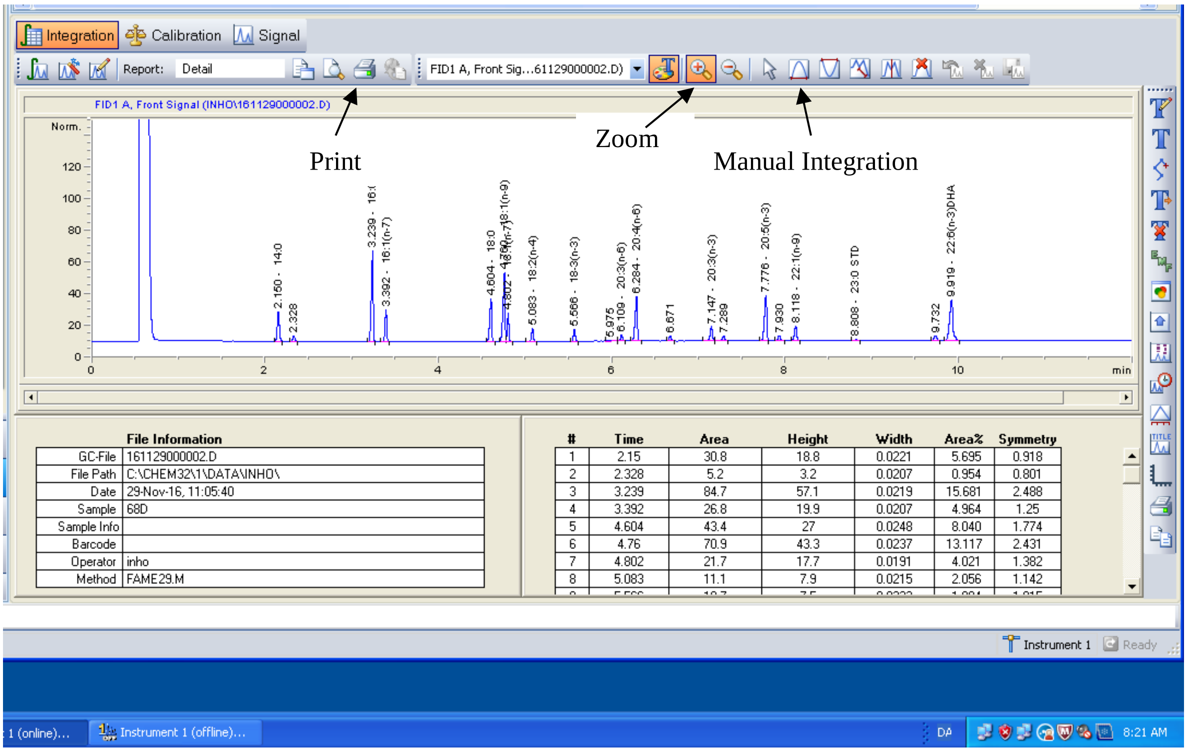

9.9 Control/correct the chromatogram (integrations and separations), and print the results.

9.10 The identification of peaks requires precise evaluation of the chromatogram, particularly for unknown or irregular samples (e.g. microbial or algal sample). Check with technicians in case of any doubt. See also **Appendix 2** regarding identification and performance check**.**

9.11 To calculate the results use Excel sheet: O:\Bioaktiv\7. Laboratorie 2.sal Lyngby\g. Regneark til analyser (excel sheets for analyses) and the file named "FAME C23 g per g". In this file calculations are based on the use of FAME C23 standard. See 11.4 and **Appendix 1** regarding calculations**.**

9.12 If the peaks get too small to obtain a proper quantification, it means that it is necessary to upconcentrate the sample by evaporation before injection (see 8.1 regarding injection volume). If this is not possible then the volume of sample can be increased from the standard volume of 0.2 µl to a maximum of 4.0 µl. If a high volume is used this can have negative effects on the chromatography and should be avoided if possible.

10. Method performance and validation

Procedure for performance check and validation is described in **Appendix 2**. We use SRM $^{@}$ 1946 (Lake Superior fish tissue) as a reference for evaluation of the trueness and the method performance in annual basis. This annual performance check includes determination of ECF as they can change as the column gets older. If ECFs are different from those of the previous year then the Excel sheet for calculation of the amount of fatty acids per g sample must be updated with the new ECF values.

11. Notes

11.1 The method description number originates from “FF analyse forskrifter”

11.2 The preparation of fatty acid methyl esters is not a part of this document. Procedure for standard methylation of fatty acids is described in analysis description no. 108. Procedure for microwave preparation of FAME is described in analysis description no. 122. Procedure for direct transesterification is described in a manuscript submitted for publication.

11.3 Fats from dairy and ruminant products contain short chain fatty acids, which are volatile and therefore require specific precautions during the FAME preparation and subsequent handling of FAMEs. Therefore, the method is not suitable for such fats. CLA cannot be discriminated from non-conjugated linoleic acid with the GC column used for this method.

11.4 If the results are intended to report as relative values (% of total FAME), input the required data including peak area for each particular fatty acid in the calculation sheet (See Excel sheet: O:\Bioaktiv\7. Laboratorie 2.sal Lyngby\g. Regneark til analyser (excel sheets for analyses) and the file named “FAME C23 g per g”). The Excel sheet is set up to exclude the internal standard in the calculation of the % fatty acid of total fatty acids. The unidentified peaks are not entered in the Excel sheet. Therefore, the sum of the fatty acids in % will not amount to 100 %. The difference between the sum of fatty acids and 100 % corresponds to the sum of unknowns in % and can be reported as “others”. If the results are intended to be reported as absolute values (mg/g sample), input required data including sample weight, internal standard area and weight as well as peak area (for each particular fatty acid) in the calculation sheet in the Excel file.

11.5 It has been shown that the alkali-catalyzed methanolysis step for the determination of long-chain fatty acids produces dimethyl and mixed-alcohol esters from the plasticizer dioctyl [actually di(2-ethylhexyl)] phthalate. An independent boron trifluoride-catalyzed methanolysis step produces a lower level of artifacts, but a two-step process produces a higher conversion than either catalyst did independently. The retention times for the dimethyl, mixed-alcohol, and di octyl phthalates may be found and pointed in relation to methyl esters of common fatty acids, See reference 3 for more details.

12. References

12.1 AOCS Official Method Ce 1b-89.

12.2 AOCS Official Method Ce 1i-07.

12.3 SHANTHA, N. C., & ACKMAN, R. G. (1991). BEHAVIOUR OF A COMMON PHTHALATE PLASTICIZER DIOCTYL PHTHALATE DURING THE ALKALI AND-OR ACID-CATALYSED STEPS IN AN AOCS METHOD FOR THE PREPARATION OF METHYL ESTERS. Journal of Chromatography, 587(2), 263–267, 263–267. doi:10.1016/0021-9673(91)85163-A

13. Safety remarks

n-Heptane

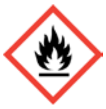

Danger

H225: Highly flammable liquid and vapour.

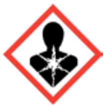

H304: May be fatal if swallowed and enters airways.

H315: Causes skin irritation.

H336: May cause drowsiness or dizziness.

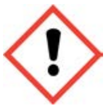

H410: Very toxic to aquatic life with long lasting effects.

EUH066: Repeated exposure may cause skin dryness or cracking.

P210: Keep away from ignition sources. No smoking.

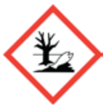

P301+310: IF SWALLOWED: Immediately call a POISON CENTER/doctor.

P273: Avoid release to the environment.

14. Handling of waste

After use the vials are placed in the waste bottle for vials in room 1.112

Appendix 1

Calculations:

1. Calculation for absolute values as fame (g/g lipids)

$$\text{Fatty acid (g/g) total lipid} = \frac{(A_S)(W_{IS})(CF_S)}{(\text{A IS})(\text{W S})(1.004)}$$

A_s= Area counts of fatty acid (x)

W_{Is}= weight of internal standard

CF_s= Theoretical correction factor of fatty acid(x)

A_{Is}= Area of internal standard

2. Calculation as relative values(% of total fatty acids)

$$\text{Fatty acid (\%)of total fatty acids} = \frac{100\ (A_x)}{(A_t - A_s)}$$

A_x= Area counts for each fatty acid x

A_t= Area counts for the rest of fatty acids

A_{IS}= Area counts for internal standard

3. Calculation as absolute values as TAG(% of total lipids)

See reference 2

Appendix 2

PERFORMANCE CHECK:

Authentic standards of FAME are used for identification and evaluation of the chromatographic separation. Fatty acids 6*c*, 9*c*, 12*c*, 15*c*, 18*c*-21:5 (21:5n-3) should be baseline separated from 23:0 (IS) and 24:0 should be baseline separated from 4*c*, 7*c*, 10*c*, 13*c*, 16*c*, 19*c*-22:6 (DHA or 22:6n-3) which should be almost baseline resolved from the 24:1 isomers. The SRM® 1946(Lake Superior fish tissue) and GLC 17 certified standard mix are used for the validation of quantifications (note 1). We use TRF/ECF for the quantifications. It is important to understand the concept and the difference (and hence the relationship) between TCF and TRF.

1- **Theoretical flame ionization response factor (TRF)** is actually the proportion (with respect to the total weight, which is the molecular weight) of the total amount of carbon atoms in the molecule (here, fatty acid methyl ester) that are responding to the FID. For fatty acids, the carbons that are responding to FID are CH, CH2, and CH3 units. Carbonyl carbon and ester carbonyls carbon do not respond to FID. So that in FAME, all the carbon atoms are active, except the ester carbon. Thus, there are N_{x-1} active carbon atoms in FAME. So that the TRF for a FAME would be:

TRF = (Nx-1) (Atomic Weight of Carbon)/MW of FAME

TRF of 23:0 FAME = 23 × 12.011/368.6312 = 0.7494

2- **The Empirical Correction Factors (ECFs)** are determined by analysis of GLC-714 at a concentration of approximately 20 mg/mL and plotted as the percent difference between the ECF and Theoretical Correction Factors (TCFs) divided by the TCFs using the standard calculation sheet (Link to the excel sheet). An example of these calculations is as follows:

% Difference = ((ECFFAMEx – TCFFAMEx)/TCFFAMEx) × 100

GLC-714 standard mix can also be used to determine if a chromatographic system is operating properly. Operation under the GC parameters listed in the method should result in a similar trend line with the long chain PUFA ECFs deviating furthest from TCFs, while the saturated and monounsaturated FAME greater than 12 carbons in length deviate from the TCFs the least. If the % Difference is greater than three for the saturated and monounsaturated FAME, it is suggested the GC parameters (e.g., detector gases, carrier gas flows, and column choice, etc.) be re-optimized to achieve a difference of 3% or less before continuing with analysis. ECFs will need to be calculated and used for the long chain PUFA (≥ 20 carbons and three double bonds).

3. **Peak identification-** The individual FAMEs are identified by their retention times, which are compared with the FAME reference standards and also reference oils (SRM© 1946). Peaks of unknown identity should *not* be included in the summation of peak areas when quantifying the concentrations of total fat, saturated, monounsaturated, polyunsaturated, EPA, DHA, other LC PUFA(Long chain poly unsaturated fatty acids) unless they have been confirmed to be fatty acids.

Note 1: The standard reference material (SRM®1946, from NIST, USA) is being used to validate the quantification procedure in annual basis. The corresponded Certified Mass Fractions (Wet-Mass Basis) for Fat and Selected Fatty Acids (as the Triglyceride) in SRM® 1946 are shown in Table 1.

Table 1. Certified Mass Fractions (Wet-Mass Basis) for Fat and selected fatty acids (as the Triglyceride) in SRM® 1946

Fat	Mass Fraction[(a)] (%)		
Fat (Extractable)	10.17	±	0.48
Fat (Sum of Fatty Acids)[(b)]	8.76	±	0.17
Selected Fatty Acids (as the triglyceride)			
Hexadecanoic Acid (C16:0) (Palmitic Acid)	1.22	±	0.04
Octadecanoic Acid (C18:0) (Stearic Acid)	0.263	±	0.011
(Z,Z)-9,12-Octadecadienoic Acid (C18:2) (Linoleic Acid)	0.348	±	0.023
(Z,Z,Z)-9,12,15-Octadecatrienoic Acid (C18:3) (Linolenic Acid)	0.221	±	0.025
Eicosanoic Acid (C20:0) (Arachidic Acid)	0.0100	±	0.0012
(Z,Z)-11,14-Eicosadienoic Acid (C20:2)	0.0990	±	0.0043
(Z,Z,Z,Z,Z)-5,8,11,14,17-Eicosapentaenoic Acid (C20:5) (EPA)	0.296	±	0.019
(Z,Z,Z,Z,Z)-7,10,13,16,19-Docosapentaenoic Acid (C22:5) (DPA)	0.335	±	0.026
(Z,Z,Z,Z,Z,Z)-4,7,10,13,16,19-Docosahexaenoic Acid (C22:6) (DHA)	0.92	±	0.10
